# Does *Acinetobacter calcoaceticus* glucose dehydrogenase produce self-damaging H_2_O_2_?

**DOI:** 10.1042/BSR20240102

**Published:** 2024-05-24

**Authors:** Victoria Lublin, Brice Kauffmann, Sylvain Engilberge, Fabien Durola, Sébastien Gounel, Sabrina Bichon, Cloée Jean, Nicolas Mano, Marie-France Giraud, Léonard Michel Gabriel Henri Chavas, Aurélien Thureau, Andrew Thompson, Claire Stines-Chaumeil

**Affiliations:** 1 Centre de Recherche Paul Pascal (CRPP), University Bordeaux, CNRS, UMR 5031, Pessac, France; 2 Synchrotron SOLEIL (CNRS - CEA), Saint-Aubin, France; 3 Institut Européen de Chimie et Biologie (IECB), Univ. Bordeaux, CNRS, INSERM, US1, UAR 3033, Pessac, France; 4 Univ. Grenoble Alpes, CNRS, CEA, Institut de Biologie Structurale (IBS), 71 avenue des Martyrs, Grenoble 38044, France; 5 Institute of Chemistry and Biology of Membranes and Nano-objects (CBMN), Pessac, France; 6 Nagoya University, Nagoya, Japan

**Keywords:** hydrogen peroxide production, protein stability, soluble glucose dehydrogenase

## Abstract

The soluble glucose dehydrogenase (sGDH) from *Acinetobacter calcoaceticus* has been widely studied and is used, in biosensors, to detect the presence of glucose, taking advantage of its high turnover and insensitivity to molecular oxygen. This approach, however, presents two drawbacks: the enzyme has broad substrate specificity (leading to imprecise blood glucose measurements) and shows instability over time (inferior to other oxidizing glucose enzymes). We report the characterization of two sGDH mutants: the single mutant Y343F and the double mutant D143E/Y343F. The mutants present enzyme selectivity and specificity of 1.2 (Y343F) and 5.7 (D143E/Y343F) times higher for glucose compared with that of the wild-type. Crystallographic experiments, designed to characterize these mutants, surprisingly revealed that the prosthetic group PQQ (pyrroloquinoline quinone), essential for the enzymatic activity, is in a cleaved form for both wild-type and mutant structures. We provide evidence suggesting that the sGDH produces H_2_O_2_, the level of production depending on the mutation. In addition, spectroscopic experiments allowed us to follow the self-degradation of the prosthetic group and the disappearance of sGDH's glucose oxidation activity. These studies suggest that the enzyme is sensitive to its self-production of H_2_O_2_. We show that the premature aging of sGDH can be slowed down by adding catalase to consume the H_2_O_2_ produced, allowing the design of a more stable biosensor over time. Our research opens questions about the mechanism of H_2_O_2_ production and the physiological role of this activity by sGDH.

## Significance

Diabetics need daily monitoring of their glycemia levels, which is achieved by using biosensors. These medical devices are equipped with active enzymes able to oxidize blood glucose, like glucose dehydrogenase (GDH). Electrons from this oxidation-reduction reaction generate a signal proportional to the quantity of substrate assayed by the enzyme. Efforts to improve enzyme stability, sensitivity, and specificity for glucose are, however, essential to increase the devices’ reliability. We have mutated soluble GDH to increase the specificity of the enzyme for glucose compared with other sugars present in the blood. Structural studies of the wild-type enzyme and mutants have pinpointed the reasons for its intrinsic instability.

## Introduction

Glucose oxidizing enzymes are oxidoreductases (EC 1.1) able to catalyze the oxidation of D-glucose (C_6_H_12_O_6_) to gluconolactone (C_6_H_10_O_6_). These oxidoreductases are divided into two main groups: glucose oxidase (GOx) [1–4], able to use oxygen as an electron acceptor, and GDHs, insensitive to oxygen. GDH are themselves subdivided into three distinct groups according to their cofactors: Flavin Adenine Dinucleotide (FAD/FADH_2_) [5,6], Nicotinamide Adenine Dinucleotide (NAD^+^/NADH) [7], or PyrroloQuinoline Quinone (PQQ/PQQH_2_) [8–10]. All glucose oxidizing enzymes can transfer protons and electrons from the hydroxyl group, carried by C1 of the β anomer of D-glucose, to their cofactor [11]. Given their basic properties, glucose oxidizing enzymes have great potential in biosensor applications for glycemic control [12]. As glucose oxidizing enzyme properties vary tremendously in terms of substrate specificity, stability, nature of electron acceptor, origin, and atomic structure, there is a scientific challenge to develop the most efficient biosensor or biofuel cell [13].

Owing to their high glucose specificity, fungal enzymes, like GOx [14] and FAD-GDH [15], as well as NAD-GDH, have been extensively used on first-generation self-test blood glucose strips. Their use in biomedical applications is, however, constrained by their low catalytic efficiency (*k*_cat_/*K*_M_ = 16 000 M^−1^·s^−1^ for GOx of *Aspergillus niger*, *k*_cat_/*K*_M_ = 20 000 M^−1^·s^−1^ for FAD-GDH of *Aspergillus flavus*), or their low activity over time, due to the difficulty of regeneration of NADH into NAD^+^ [16]. To overcome these limitations, soluble glucose dehydrogenase (sGDH) from *Acinetobacter calcoaceticus* has been widely studied and used as an anodic enzyme in biofuel cells, taking advantage of its PQQ cofactor firmly bound in its active site (*K*_d_ = 3.10^−8^ mM), its high turnover (approximately 8000 s^−1^) and insensitivity to molecular oxygen [17,18]. Consequently, due to its insensitivity to O_2_ and high turnover number, sGDH is better than GOx to avoid electron leakage, via O_2_, during glucose monitoring, and to have quicker results. However, this dimeric enzyme, composed of two identical 453 amino acid monomers (pI = 8.75), also presents drawbacks in terms of broad substrate specificity and loss of activity over time. The lack of specificity of these enzymes for glucose has led to imprecise blood glucose measurements in patients under treatment [19,20]. From 1997 to 2009, the Food And Drug Administration received several reports of death after glycemia measurements of 3 to 15 times higher than the real value, because of the detection of maltose or other sugars [21]. Molecular engineering studies on sGDH are essential to improve the reliability and safety of the devices composed of these enzymes.

Several sGDH optimization strategies, using site-directed mutagenesis, have been previously reported. To increase enzyme stability, the residues selected for substitutions were located at the dimer interface to improve the stability of the quaternary structure of the dimer; either by introducing a disulfide bridge (S391C) [22] or by increasing the number of hydrophobic residues (N316F/Y394F, N316F/Y394I, and T392V/T393V) [23] at the dimeric interface. To influence substrate specificity, targeted residues were localized at, or near, the active site. N428T [24] and D143E [25] mutations significantly reduced selectivity for lactose and maltose, with a cumulative effect for the double mutation D143E/N428T [26]. The N428C mutant, characterized by Durand *et* *al*. in 2010, doubled the steady-state catalytic rate constant (*k*ss ≈ 8 000 s^−1^) when compared with the wild-type enzyme (*k*_ss_ ≈ 4 200 s^−1^) for 50 mM of glucose [27]. *In silico* modelling revealed the eventual elimination of a hydrogen bond between the sulfoxide group of C428 and the hydroxyl group of Y343, creating a more favorable environment for PQQ insertion in the active site.

In this work, structure/function relationship studies of mutated sGDH (Y343F and D143E/Y343F), selected for their higher substrate specificity to glucose, allowed us to infer direct or indirect hydrogen peroxide (H_2_O_2_) production by sGDH. Direct consequences on enzyme functionalities are (i) the cleavage of PQQ and (ii) the loss of activity toward glucose oxidation. Initial experiments have been conducted to protect the enzyme from auto-degradation by the H_2_O_2_ produced *in situ*, and consequently improve its stability.

## Results

### Steady-state kinetic parameters for wild-type and mutants

**Figure 1:**
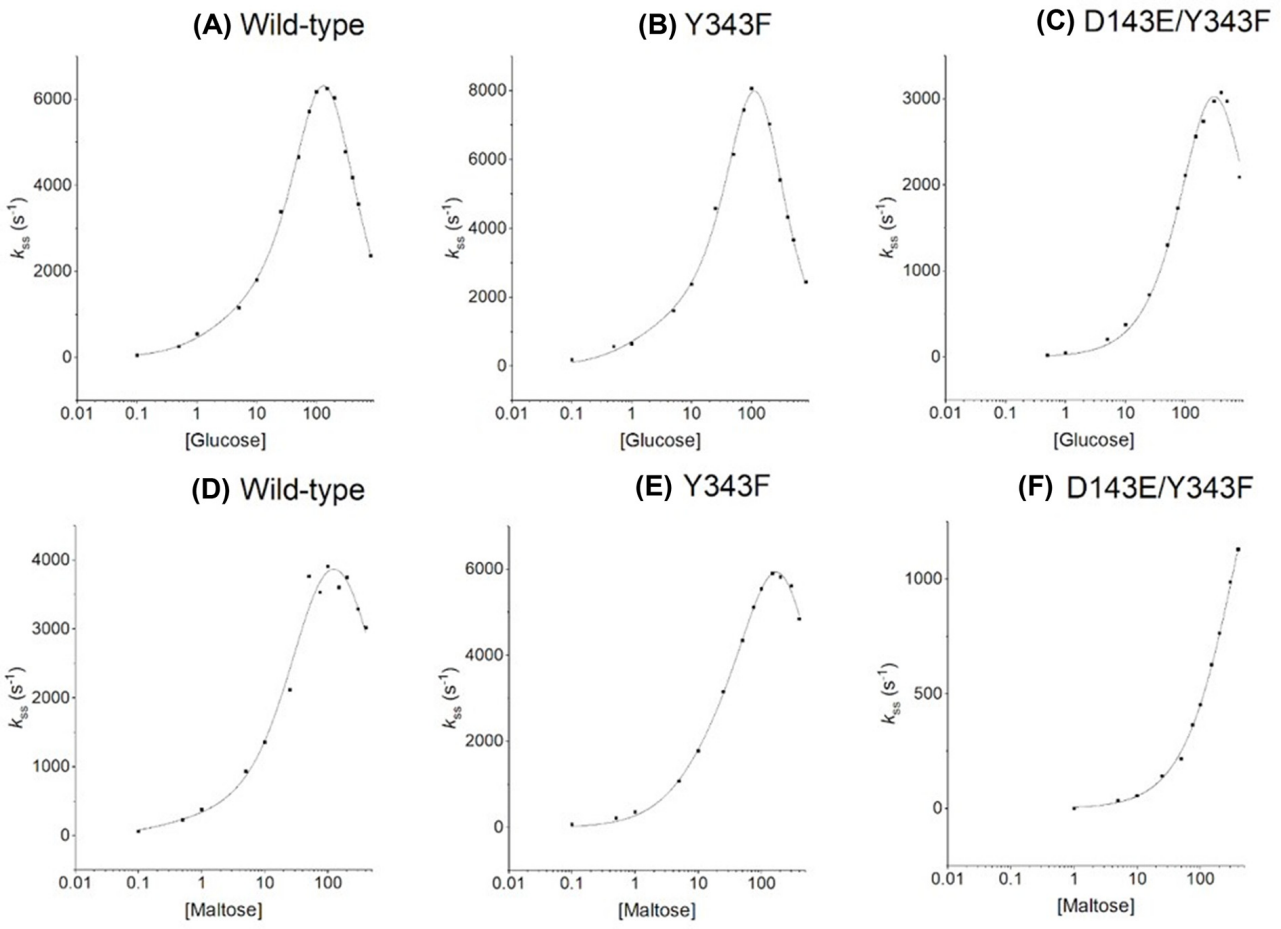
Non-linear regression on glucose and maltose range for wild-type sGDH and Y343F or D143E/Y343F mutants at 37°C The oxidation activities of sGDH wild-type and mutants were determined by spectrometry to follow the reduction of 2,6-dichlorophenolindophenol (DCIP) (ε_600_ = 21.6 mM^−1^·cm^−1^) at 600 nm, using phenazine methosulfate (PMS). Sugar concentrations varied between 0 and 400 mM for maltose and 0 and 800 mM for glucose. Curves in A, B, D, E were fitted according to (eqn 1); curve C according to (eqn 2) and curve F according to (eqn 3) (see Material and Methods section).

Steady-state kinetic parameters were determined for all three enzymes (wild-type, Y343F, and D143E/Y343F mutants) with D-glucose and D-maltose, using 2,6-dichlorophenolindophenol (DCIP) and phenazine methosulfate (PMS) as electron acceptors. Non-linear regression was applied to experimental data and results are shown in Figure 1. Fitting equation [28] was applied to the measurements and led to the constants summarized in Supplementary Table S2. The wild-type and the Y343F mutant sGDH show negative cooperativity in the oxidation of glucose and maltose, with two Michaelis–Menten constants and a large increase between *K*_M1_ versus *K*_M2_. The D143E/Y343F mutant presents a classical Michaelian behavior with both monomer sites equivalent and a single value of *K*_M_. At low sugar concentrations, the mutants tend to show an improvement in glucose over maltose selection. For example, at physiological concentrations (5 mM), the Y343F and the D143E/Y343F mutants have a glucose oxidation steady-state catalytic rate constant (*k*ss) 1.5 and 7.1 times increased compared with that of maltose, respectively (see Table 1). In contrast with the wild-type, this corresponds to an increase in glucose selectivity of 1.2 and 5.7 times for the Y343F and D143E/Y343F mutants, respectively. As for the wild-type enzyme, mutants are inhibited by an excess of glucose.

**Table 1:** Steady-state rate constant ratio of sGDH wild-type and mutants (Y343F or D143E/Y343F) toward glucose (Glc) or maltose (Mal) evaluate the benefits of the mutated enzyme (MUT) compared with wild-type (WT)

	Selectivity Glc/Mal			Selectivity Glc/Mal compared with WT	
	*k*ssGlc/*k*ssMal			(*k*ssGlc/*k*ssMal Mut)/(*k*ssGlc/*k*ssMal WT)	
[Oses] mM	WT	D143E/Y343F	Y343F	D143E/Y343F	Y343F
0.1	0.80	n.d.	2.51	n.d.	3.13
0.5	1.12	n.d.	2.62	n.d.	2.33
1	1.43	n.d.	1.79	n.d.	1.25
5	1.24	7.11	1.50	5.74	1.21
10	1.33	7.95	1.34	5.97	1.00
25	1.60	6.08	1.45	3.81	0.91
50	1.24	7.12	1.42	5.75	1.14
75	1.62	5.66	1.45	3.50	0.90
100	1.58	5.56	1.46	3.52	0.92
150	1.73	4.87	n.d.	2.81	n.d.
200	1.61	4.27	1.21	2.66	0.75
300	1.45	3.59	0.96	2.47	0.66
400	1.39	3.24	0.89	2.34	0.64

n.d., not determined.

### Structural studies of sGDH mutant crystals

**Figure 2:**
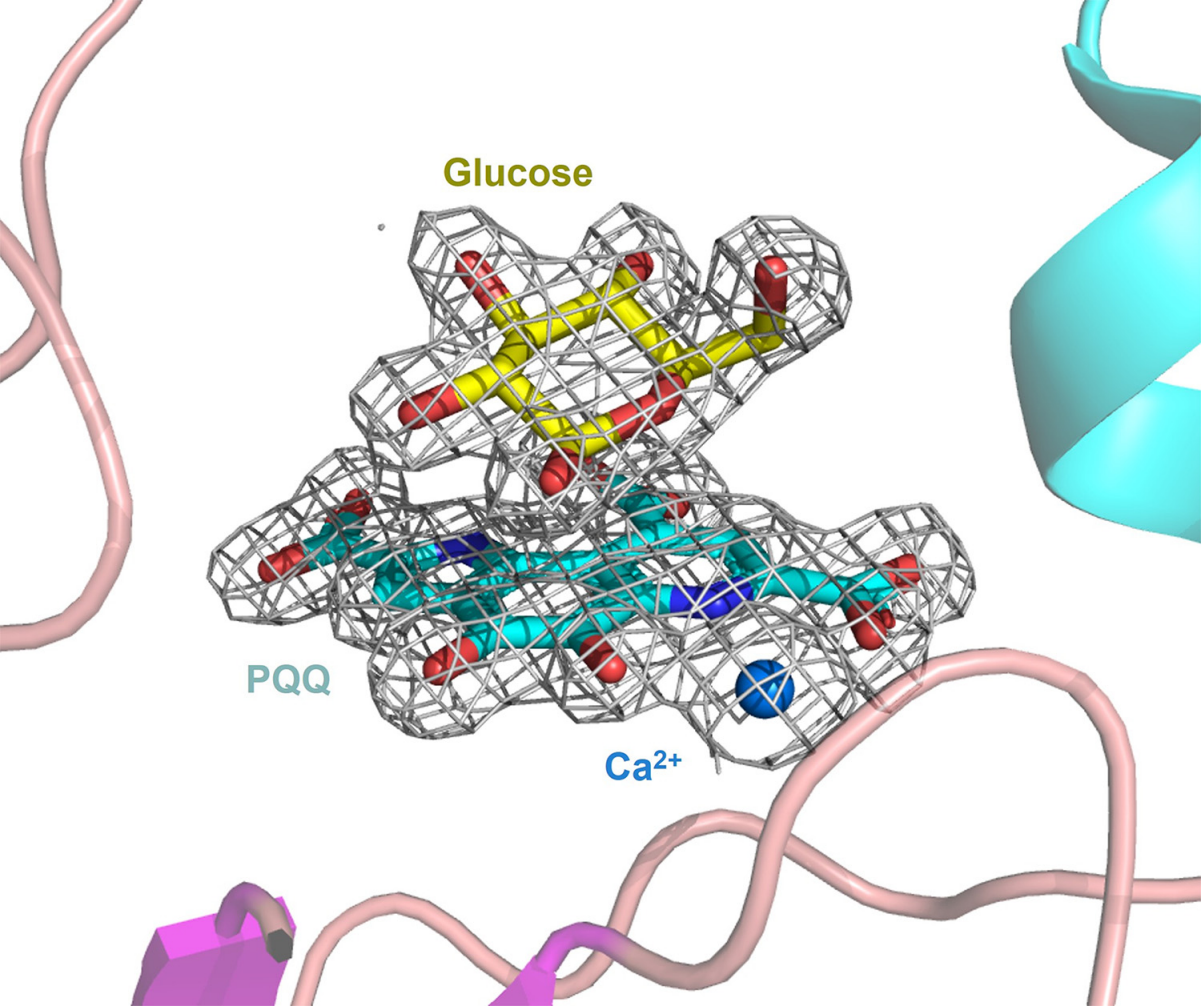
Active site of the holo sGDH wild-type with glucose The figure was prepared by the authors based on the structure factors and refinement, published by Oubrie *et al*. in 1999 (PDB 1CQ1) [30], obtained from the PDB-REDO [31] server. The electron density (grey mesh) shown is calculated from a Polder Omit Map (PHENIX) contoured with a cutoff of 5σ. The positions of the protein chain (ribbon diagram), calcium atom (dark blue), PQQ (blue ball and stick model), and glucose binding (yellow ball and stick model) are shown.

The first crystal structures of sGDH from *A. calcoaceticus* were reported by Oubrie et al. [8,29], in which structures of holo-sGDH, both alone and in complex with glucose, were obtained by soaking apo-crystals with PQQ and/or glucose (Figure 2). These soaking experiments provoked structural rearrangements in the crystals that caused a change in color and crystal space group and frequently resulted in severe crystal cracking (A. Oubrie, *personal communication*), making it difficult to obtain well-diffracting crystals. In the current study, a different approach was taken whereby active holo-sGDH was reconstituted before crystallization trials [10].

Crystals of the wild-type, Y343F, and D143E/Y343F mutants of sGDH were prepared for X-ray diffraction studies and structure determination (see details in Supplementary Table S1).

**Figure 3:**
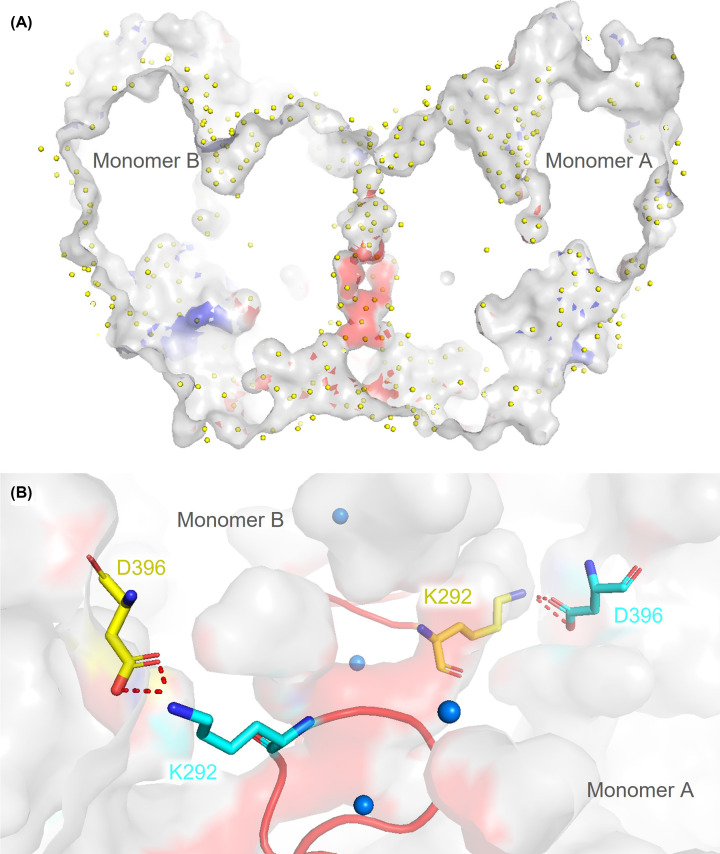
Dimeric interface of the wild-type sGDH (**A**) The electrostatic charge was calculated with the macromolecular electrostatics calculation plugin Plug-APBS available under PyMOL [33]. A well ordered and extensive water network (yellow spheres) is present between the two monomers, as seen here in a lateral view of the dimer interface. (**B**) Residues K272 and D396, from opposing subunits, form a salt bridge, identified with PISA interface [32], a web-server available via PDBe. The residues in blue belong to subunit A, those in yellow to subunit B. The region between residues N262 and D273, referred to as the 4CD loop (red), is stabilized by calcium ions (blue) present at the dimeric interface.

Briefly, all crystal structures contained a tight dimer of sGDH in the asymmetric unit, the dimeric interface extending over 1110 Å^2^ [32] being maintained by the presence of two calcium ions as described in Oubrie *et al*. [30] interacting with the N262-D273 loop, referred to as the 4CD loop in the nomenclature of Oubrie *et al*. (Figure 3). The organization of the protein chain around these calcium ions allows the formation of salt bridges between K272 and D396 of each monomer. In addition, the presence of two cis-peptide bonds between W265 and P266, and between L324 and Y325, serve to rigidify the dimer interface and orient aromatic side chains to best stabilize the dimer (see Supplementary Figure S1). The dimer interface is further stabilized by an extensive hydrogen bonding network of 15 bonds and a striking network of ordered water molecules which is conserved in all structures (Figure 3). The B factors of the structures typically average at approximately 30 Å^2^; however, the electron density is poor or absent for two loops (S105-L110 and P336-Y343, the latter of which contains the single disulfide bridge per monomer). It is noteworthy that the Y343F mutation is found in this loop, which is near to and covering the active site. The electron density is difficult to model where the loop is disordered, an indication that this part of the enzyme remains particularly flexible and can adopt different conformations. It is interesting to note that the L324-Y325 cis-peptide bond immediately precedes the latter loop and may act as a stable basis for loop movements so that these do not result in the reorganization of the active site or dimer interface. We also note the proximity of the W265-P266 cis-peptide to the active site and speculate that this may help stabilize the fold in this region (see Supplementary Data S3). Coupled size exclusion chromatography and small angle scattering measurements (SEC-SAXS) reveal solution scattering curves coherent with the crystal structure for wild-type and both mutant structures (see Supplementary Figure S10).

### Unexpected cleavage of the PQQ was observed in all structures

**Figure 4:**
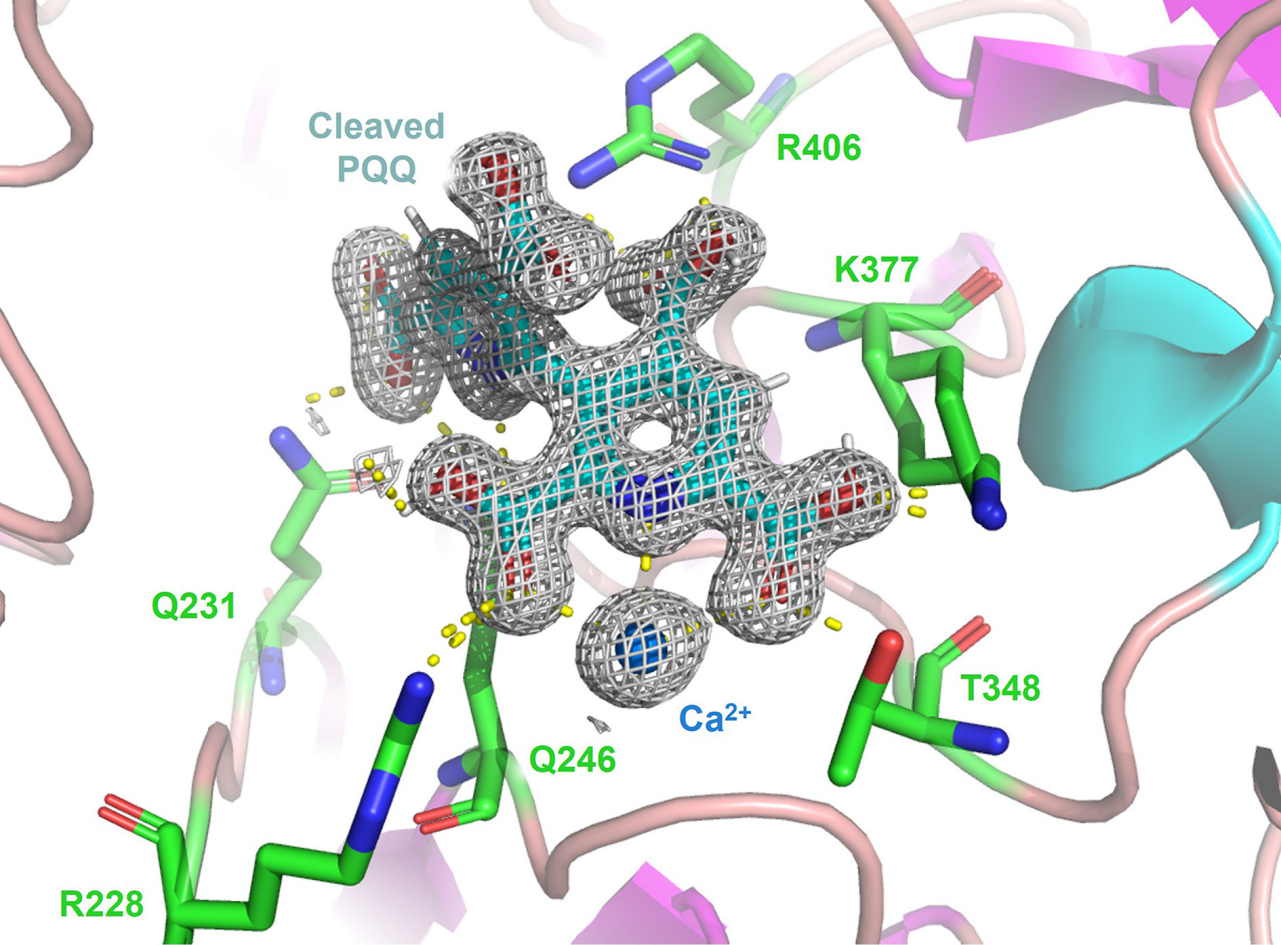
Active site of the holo sGDH wild-type The active site of PQQ from the wild-type enzyme at pH 8. The density at 5 σ from a Polder Omit Map (PHENIX) is superimposed onto a model of cleaved PQQ. Residues close to the cofactor are labeled, and show little reorganization compared with the structures published by Oubrie *et al*. When compared with the structure in Figure 2, the cleavage of PQQ is clearly demonstrated and the pyrrole ring is seen to be almost perpendicular to the quinone. Similar cleavage was observed in all measured structures in this study. Electron density is observed directly above the cleaved quinone, which could possibly be attributed to a molecule of H_2_O_2_, although there is no direct evidence to substantiate this hypothesis. Consequently, this density has intentionally been left unmodeled in the refined structure.

2*F*_obs_-*F*_calc_ electron density maps from the wild-type and both mutant structures all showed a different molecule instead of the PQQ-cofactor, corresponding to a PQQ modified cofactor, with the bond between C4 and C5 missing (Figure 4 and Supplementary Movies S1, 2 and 3). The absence of this C-C bond permits a large rotation of the pyrrole ring (110° ± 2°) to assume a completely different conformation. Calculation of Polder omit maps (Figure 4) strongly support this interpretation of the electron density [34]. Numerous attempts to co-crystallize mutant protein with glucose and maltose substrates all failed to locate the sugar substrates in the resolved structures (data not shown). Indeed, as can be seen in Figure 4, the rotation of the pyrrole ring obscures the glucose binding site observed by Oubrie [8]. Interestingly Winkler *et al*. [35] describe an oxidation product of free PQQ when exposed to H_2_O_2_ in acidic conditions. This reported oxidation product (pyrrolo-pyridine) assumes the same conformation as the one observed in the current crystal structures and, moreover, does not reveal the well-known UV-visible spectroscopic signals expected from oxidized or reduced forms of PQQ (see Supplementary Figure S2).

The natural PQQ shows a characteristic UV-fluorescence signal at 340 nm, a signal that was lost for the crystallized protein when compared to the fresh enzyme (see Supplementary Figure S3). Mass spectrometry and NMR measurements (see Supplementary Figure S4) on the stock of PQQ used in these studies confirmed that the PQQ used to reconstitute the active form of sGDH was not damaged. Taken together, these results demonstrate that the cleavage of the PQQ was not the result of mistreatment during the sample preparation phase.

### Can the cleavage of the PQQ be attributed to X-ray-induced damage?

Because of the damaging effects of X-ray radiation, we intended to clarify whether the irradiation of the crystals could be at the origin of the cleavage of the PQQ molecule. The size of the crystals used for data collection (measuring 250 × 150 × 150 µm^3^ for the wild-type, 100 × 75 × 60 µm^3^ for the mutant Y343F and 130 × 90 × 90 µm^3^ for the mutant D143E/Y343F) was larger than the beam footprint used for the mutant crystals (40 × 20 µm^2^). The crystals were subjected to a relatively modest dose of X-ray during data collection, estimated (from the measured beam flux, the exposure time and the crystal volume) as 2.75 MGy for the single and double mutant crystals and 0.9 MGy for the very high-resolution wild-type crystal [36]. These values should be read in context with the estimated radiation dose limit for frozen crystal, 30 MGy, above which the crystal structure becomes significantly modified [37]. Second, the electron density based on refinement with BUSTER ‘early’ and ‘late’ dose stage Fourier syntheses [38] (see Supplementary Figure S5) demonstrated the same cleavage of the cofactor. Third, a data set of the wild-type recorded on a rotating anode X-ray generator at a slightly lower resolution, on a similar crystal and an absorbed dose of around 0.1 MGy, displayed the same bond cleavage (see supplementary data Figure S6B). Finally, UV-visible spectroscopy measurements performed on melted crystals that had not been exposed to X-ray also showed a lack of spectroscopic signals for PQQ (see above and Supplementary Figure S4), suggesting that the cofactor was cleaved prior to X-ray exposure. Taken together, these points strongly suggest that the cleavage of PQQ is not caused by direct radiation damage by the X-ray beam. The possibility for indirect radiolysis (production of reactive fragments by very low dose X-ray irradiation, or by potential contamination in the crystallization conditions) cannot, however, be completely ruled out.

### Does sGDH produce H_2_O_2_?

**Figure 5:**
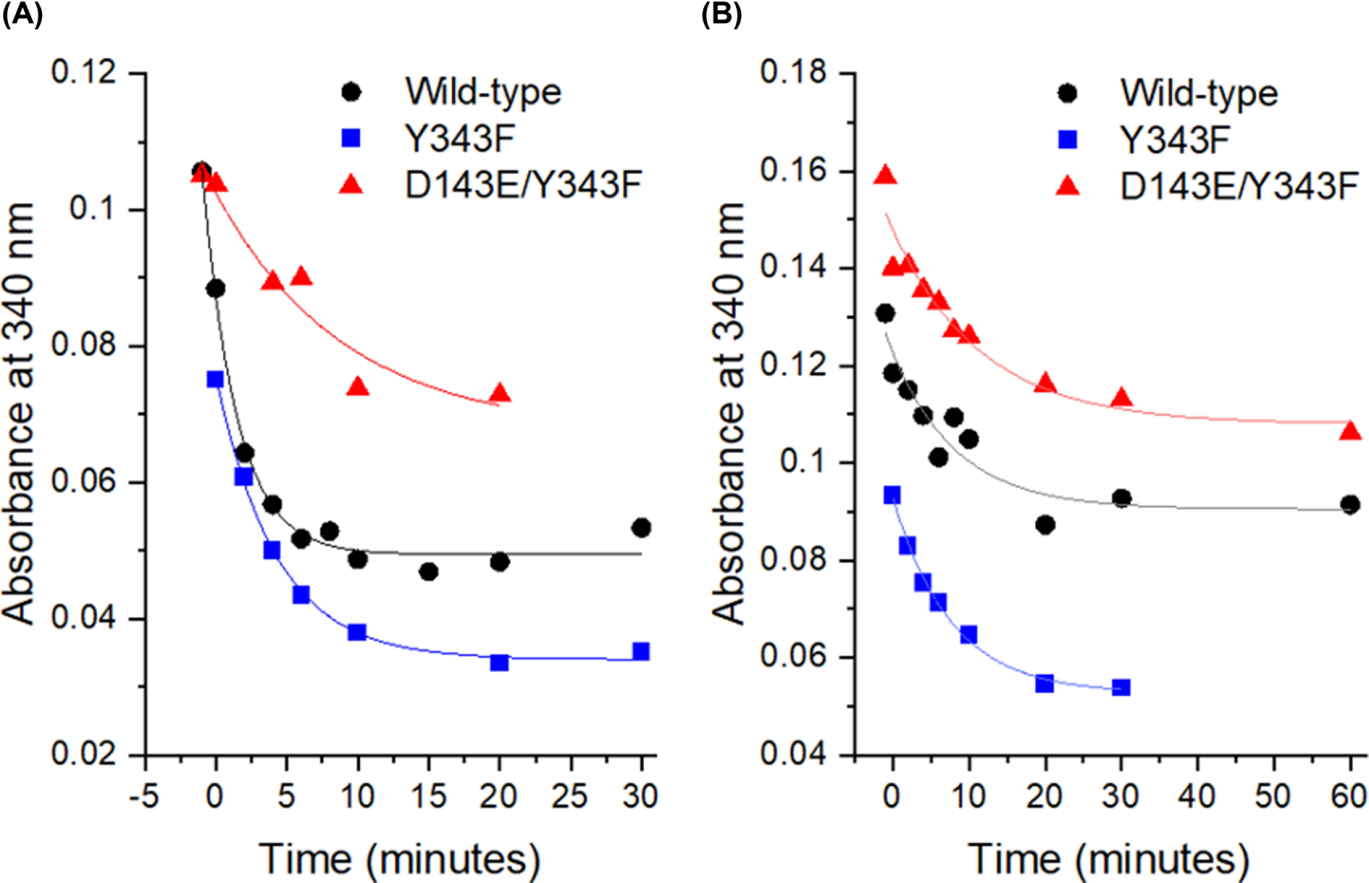
Sensitivity of sGDH to H_2_O_2_ at pH 7.5 (A) and pH 5.0 (B) The *k*_obs_ were calculated from the decreasing exponential fit at pH 7.5 and at pH 5.0; values are reported in Table 2.

Spectroscopy was used to measure the absorbance at 340 nm and consequently demonstrate the sensitivity of the holo-enzyme (wild-type, Y343F and D143E/Y343F mutants) (Supplementary Figure S7) or of the isolated PQQ to H_2_O_2_ (Supplementary Figure S8). After the addition of 25 mM of H_2_O_2_ to a solution of enzyme at 7.5 µN in site (1 µM of dimer corresponds to 2 µN of active site or monomer), the absorbance at 340 nm follows an exponential decrease over time (Figure 5 and see Supplementary Figure S7). This absorbance decrease is observed for all three mutant enzymes at pH 7.5 (Figure 5 A), and at pH 5.0 (Figure 5 B). The signal is halved in less than 10 minutes for each, at pH 5.0 and at pH 7.5. Observed rate constants and half-live are summarized in Table 2. This diminution of absorbance at 340 nm for the enzymes in solution is identical to that measured on the dissolved crystals (see Supplementary Figure S3), suggesting that the enzyme’s cofactor may have been damaged by the presence of H_2_O_2_ or another oxidizing agent directly in the crystallization droplet.

**Table 2:** Observed rate constant and half life of absorbance at 340 nm after exposition of sGDH to H_2_O_2_ at pH 7.5 and pH 5

	sGDH WT	sGDH Y343F	sGDH D143E/Y343F
pH 7.5			
*k*_obs_ (s^−1^)	0.42 ± 0.04	0.24 ± 0.02	0.11 ± 0.07
*t*_1/2_ (min)	1.66 ± 0.18	2.94 ± 0.20	6.28 ± 3.68
pH 5			
*k*_obs_ (s^−1^)	0.12 ± 0.04	0.13 ± 0.01	0.09 ± 0.02
t_1/2_ (min)	5.86 ± 1.93	5.27 ± 0.49	8.11 ± 2.17

If sGDH could produce H_2_O_2_ in its holo-form, such a mechanism would account both for the bond breakage in PQQ and for structural reorganizations within the enzyme giving rise to the appearance of the crystals. Further experiments were then designed to explore this hypothesis. To identify the origin of H_2_O_2_ production, experiments were carried out in the presence of an enzymatic system coupled to myeloperoxidase from *Rhodopirellula baltica* (*Rb*MPO) and sGDH at 37°C, in the presence of aminophenyl fluorescein (APF). In this reaction, if an increase of fluorescence emission is measured at 525 nm (λ_ex_ = 485 nm), it would be directly proportional to the quantity of fluorescein, which is, in turn, proportional to the quantity of H_2_O_2_ produced by sGDH which has been converted in HOCl by *Rb*MPO. The glucose oxidase from *Aspergillus niger* (*An*GOx), known to produce H_2_O_2_, was used as a positive control to confirm that this enzymatic coupled system works under our experimental conditions. The coupled enzyme system was tested at pH 7.5 on the wild-type, Y343F and D143E/Y343F enzymes, for the holo-forms in the presence or absence of glucose (Table 2). For the holo-forms, we observed significant production of H_2_O_2_ (*P*-value < 0.05). The addition of 250 mM glucose permits to increase between 2 and 4 times the production of H_2_O_2_. The observed H_2_O_2_ production results may, therefore, be underestimated for the holo-forms. Additionally, isolated PQQ is not a source of H_2_O_2_.

### Consequences of H_2_O_2_ production on sGDH

**Figure 6:**
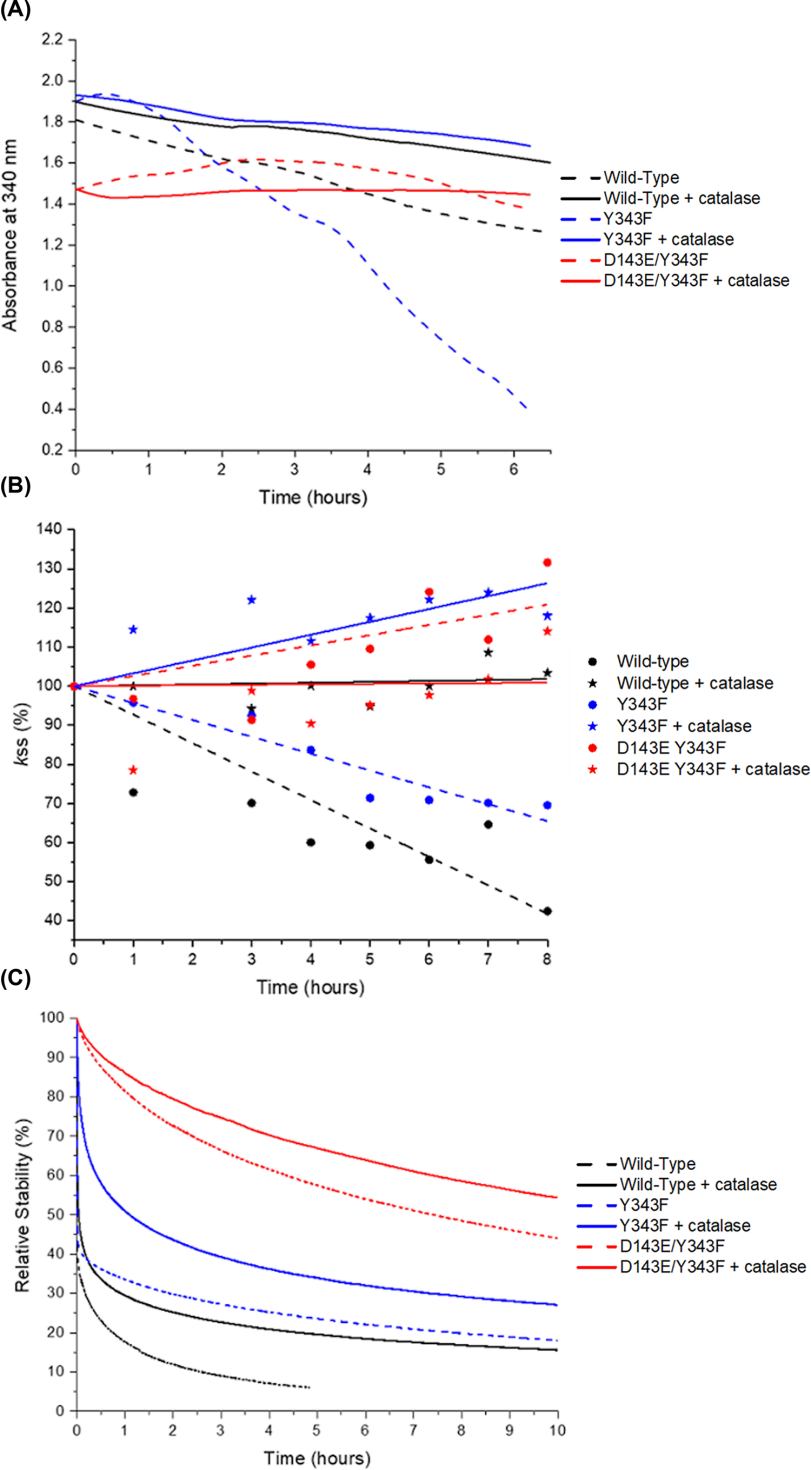
Evidence of aging and catalase effect Evidence for the aging of sGDH at 25°C, observed through three different parameters: spectroscopic properties at 340 nm (**A**), retention of activity (**B**), and the relative stability of modified electrodes measured using chronoamperometry at +0.3V vs. Ag/AgCl in a 50 mM TRIS buffer solution (pH 7.5) containing 50 mM glucose under air at 25°C (**C**). The time-dependent changes are shown in the presence (indicated by bold lines) or absence (represented by dashed lines) of 10,000 U catalase. The figures maintain the same color scheme throughout the three graphs.

To assess whether the production of H_2_O_2_ directly impacts the activity of sGDH and the integrity of PQQ (Figure 6A), we monitored the stability of glucose oxidation activity (Figure 6 B) and the spectral characteristics of PQQ over time, without any exogenous addition of H_2_O_2_. A notable decline was observed in the absorbance at 340 nm, indicative of enzyme activity, with a 5-fold decrease for the Y343F mutant and a 1.4-fold decrease for the wild-type enzyme over 6 h. This reduction in spectral signal aligns with the absence of a UV-visible spectroscopic signal characteristic of cleaved PQQ, as previously described by Winkler *et al*. [35], suggesting the influence of endogenously produced H_2_O_2_ on sGDH (Figure 5). Concurrently, a reduction in glucose oxidation activity, quantified by the rate constant (*k*ss), normalized to 100% at *t* = 0 for each enzyme variant (Figure 6B), was documented. Specifically, activity losses of 60% for the wild-type and 30% for the Y343F mutant were recorded after 8 h at 25°C. Notably, alterations to the disulfide bridge were not detected using dithionitrobenzoate (DTNB), a reagent selectively reactive toward free thiol groups (see Supplementary Data S9). This finding suggests that the observed decline in enzymatic activity is not attributable to disulfide bridge disruption. Given the oxidizing environment within the enzyme and the protective nature of the disulfide bond, it is plausible to infer that the compromised glucose dehydrogenase activity results from the oxidative damage to the quinone moiety by H_2_O_2_, rather than alterations in the disulfide bridge. At pH 7.5, the addition of catalase slows down the aging of the enzyme. By consuming the H_2_O_2_ once it is produced, catalase indirectly maintains the integrity of the cofactor and compared with the enzyme without catalase, increases the retention of enzymatic activity of sGDH for at least 8 h. This catalase activity is also an indirect confirmation of the production of H_2_O_2_ and limits the consequences on the cleavage of PQQ by trapping the H_2_O_2_ produced by the enzyme. For the D143E/Y343F mutant, these observations are less clear; this enzyme was naturally more stable as shown in Figure 6B,C (red curve dotted line) and may have been less impacted by H_2_O_2_ damage to PQQ, so the addition of catalase does not change the retention of activity results at 8 μN of enzyme in 50 mM TRIS pH 7.5 buffer at least during the 8 h of measurements. The electrochemical stability of the modified electrodes was assessed using chronoamperometry at +0.3V vs. Ag/AgCl in a 50 mM TRIS buffer solution (pH 7.5) containing 50 mM glucose under air at 25°C, with the presence of 10,000 U catalase indicated by bold lines and its absence shown as dashed lines. To prevent the reduction of O_2_ to H_2_O_2_ on the osmium complexes, the PAA-PVI-[Os(4,4ʹ-dimethyl-2,2ʹ-bipyridine)2Cl]^+/2+^ with an apparent redox potential of +0.1V vs. Ag/AgCl was chosen [39,40]. Figure 6C demonstrates that, for each enzyme, the stability is superior in the presence of catalase compared with its absence, implying the generation of H_2_O_2_ during the reaction over time. The trend of stability obtained through amperometry aligns with the data presented in Figure 6 from a homogeneous solution.

## Discussion

Soluble glucose dehydrogenase is an enzyme with high potential value for use in biosensors, due to its lack of sensitivity to O_2_, and its rapid turnover for both monomers, demonstrated by a *k*_cat1_ of 1 170 s^−1^ and *k*_cat2_ = 25 840 s^−1^ respectively, for glucose and its use of the prosthetic group PQQ (rather than a cofactor which has to be continually renewed). On the other hand, sGDH has a relatively poor substrate specificity, binding a number of common blood sugars. A glucose-specific form would be of high value for monitoring blood sugar levels in diabetes. We have identified point mutations that have been implemented in singly or doubly mutated enzymes (Y343F and D143E/Y343F) and which demonstrate, at physiological concentration, a selectivity for glucose 1.5 (Y343F) and 7.1 (D143E/Y343F) times higher compared with maltose (Table 3).

**Table 3:** H_2_O_2_ production by wild-type, Y343F mutant, and D143E/Y343F mutant sGDH

	Controls				WT		Y343F		D143E/Y343F	
**PQQ**			+							
**sGDH holo**					+	+	+	+	+	+
***Rb*MPO**		+	+	+	+	+	+	+	+	+
***An*GOx**				+						
**Glucose**				+		+		+		+
**NaCl**	+	+	+	+	+	+	+	+	+	+
**APF**	+	+	+	+	+	+	+	+	+	+
**Buffer**	+	+	+	+	+	+	+	+	+	+
**IF (AU)**	6.04E+05	5.49E+05	1.42E+06	3.24E+07	1.23E+07	5.01E+07	1.01E+07	4.33E+07	2.60E+07	4.34E+07
**Standard deviation IF (AU)**	8.18E+04	1.05E+04	5.03E+05	3.67E+06	7.09E+05	5.26E+06	8.71E+05	3.58E+06	3.25E+06	1.14E+05
**equivalent quantity H_2_0_2_ (pmol)**	0.20	0.19	0.48	10.99	4.17	16.99	3.43	14.67	8.82	14.71
**Standard deviation (pmol)**	0.03	0.00	0.17	1.24	0.24	1.78	0.30	1.21	1.10	0.04
**Test ANOVA pvalue**		3.11E-01	5.79E-02	1.14E-04	9.15E-06	9.15E-06	4.67E-05	3.25E-05	6.12E-10	2.18E-05
Significant (*) or non significant (ns)		ns	ns	*	*	*	*	*	*	*

Experiments were realized in 50 mM sodium phosphate buffer pH 7.5 at 37°C.

Structural studies of these mutants revealed a cleaved cofactor: a spectroscopically silent molecule that has previously been identified as a reaction product of PQQ with H_2_O_2_ under acidic conditions. By monitoring the spectroscopic signature of PQQ in the reconstituted enzyme, both in solution and in crystalline forms, we observed significant cleavage of PQQ. This observation indicates alterations in the structure and functionality of the enzyme, potentially linked to the enzymatic process. While this cleavage of PQQ suggests oxidative stress within the microenvironment of the enzyme, it is crucial to note that additional experiments within this study provide supportive evidence suggesting that H_2_O_2_ may be involved, directly or indirectly, as a byproduct of the catalytic process. H_2_O_2_ can then cleave the PQQ in the active site with consequences for the integrity of the PQQ in the active site and the stability of the enzyme. We have furthermore demonstrated the retention of the spectroscopic signature from undamaged PQQ and the glucose oxidation activity, after incubation of the enzyme with catalase at pH 7.5. This observation provides additional support for the hypothesis of the enzyme generating free H_2_O_2_, as catalase’s protective activity against free H_2_O_2_ is evident both in solution by following the activity, and through electrochemical measurements for enzymes wired on the surface of an electrode.

One of the great strengths of X-ray crystallography is that, although the derived protein structure corresponds to an atomic model based on the observed electron density, the electron density shows what is present in the structure or what is missing from the structure. Hence, there is no reason to question the observation of cleaved PQQ in the crystal structure: the electron density at high resolution is unambiguous. We have also demonstrated, by spectroscopic means and by low-dose data collection, that this cleavage is unlikely to be caused by direct X-ray damage.

Questions remain as to why sGDH produces H_2_O_2_ (or indeed if it does so in a cellular environment), and by what reaction mechanism. Indeed, inactivation of this mechanism by mutation, if this can be achieved without abrogation of the dehydrogenase activity, would be an interesting means of producing a more stable enzyme for medical usage. Whilst these questions are not answered by the current study, a number of potentially significant observations have been made that offer further opportunities for investigation. We hope to address these points in complementary studies.

In conclusion, what we envisaged to be an investigation of the structural basis of increased selectivity of mutant sGDH for glucose has serendipitously revealed a complicated but rich opportunity to study the activity of this enzyme.

## Materials and methods

### Chemicals and materials

PQQ, D(+)-glucose, TRIS buffer, CaCl_2_, DCIP, PMS, APF, *An*GOx, catalase and other chemicals were purchased from Sigma and used as received except for *An*GOx that is purified at homogeneity as described by Courjean *et al*. [41].

### Strains and cell-culture conditions

The bacterial strains and plasmids used in this work are summarized in Table 4. *Escherichia coli* strain DH5α was purchased from New England BioLabs® and was used to amplify the plasmids. BL21(DE3)Star pLysS cells were used to over-express proteins. *E. coli* strains (DH5α and BL21(DE3)Star pLysS) were cultivated in Luria-Bertani (LB) medium supplemented with 100 µg/ml ampicillin and 17.5 µg/ml chloramphenicol at 37°C and 190 rpm. Plasmid pgp492 Wild-type bearing the sequence of the soluble sGDH of *A. calcoaceticus* was obtained from N. Goosen [42]. Plasmids pgp492 Y343F and pgp492 D143E/Y343F bearing the sequence of the sGDH of *A. calcoaceticus* mutated in position 143 or 143 and 343, respectively, were constructed as described below.

**Table 4:** Strains and plasmids

Strains and plasmids	Genotype	Source
*Strains of E. coli*		
NEB5α	F-, *fhuA2, D(argF-lacZ)U169, phoA, glnV44, f80D(lacZ)M15, gyrA96, recA1, relA1, endA1, thi-1, hsdR17*	New England BioLabs® Inc
BL21(DE3)Star pLysS	F-*omp*T *hsd*SB (r_B_-, m_B_-) *galdcmrne*131 (DE3) pLysS (Cam^R^)	ThermoFisher Scientific
*Plasmids*		
pGP492 Wild-Type	Amp^r^ *lacZ*, native soluble glucose dehydrogenase from *Acinetobacter calcoaceticus* under control of the strong lac promoter	Cleton-Jansen *et al*. 1989
pGP492 Y343F	Amp^r^ *lacZ*, Y343F mutant soluble glucose dehydrogenase from *Acinetobacter calcoaceticus* under control of the strong lac promoter	This work
pGP492 D143E/Y343F	Amp^r^ *lacZ*, D143E/Y343F mutant soluble glucose dehydrogenase from *Acinetobacter calcoaceticus* under control of the strong lac promoter	This work

Amp^r^, ampicillin resistance.

### Cloning of the sGDH mutants

The mutations were introduced in sGDH following the instructions given by the QuickChange method. Primers 5′-ccttcatcaaaagagcatcagtcaggtcgtc-3′ (forward) and 5′-gacgacctgactgatgctcttttgatgaagg-3′ (reverse) were used to introduce the mutation in position 143. Primers 5′-gtggagagatgaccttcatttgctggccaac-3′ (forward) and 5′-gttggccagcaaatgaaggtcatctctccac-3′ (reverse) were used to introduce the mutation in position 343. The reaction was done in a thermal cycler apparatus (BioRad T100) for 30 cycles of 1 min at 98°C, 30 s at 52°C, and 4 min at 72°C. The PCR product was digested overnight by *Dpn*I to eliminate the parental plasmid and then transformed into DH5α bacteria. Clones were selected on LB medium supplemented with ampicillin and chloramphenicol. The mutated plasmids were then purified with a Qiagen kit and sequenced by Genewiz® (Azenta Life Sciences).

### Expression, purification, and reconstitution of the active sGDH wild-type Uniprot code P13650 and mutants

Production, purification, and reconstitution (PQQ 80198, Sigma) protocols of the enzyme are based on previously published data [10]. Briefly, apo enzymes were purified from *E*. *coli* BL21 (DE3) strain transformed by pgp492 WT or mutated versions of the sGDH open reading frame by cation exchange chromatography on source 30S column due to the high isoelectric point of sGDH. The holo-enzyme was prepared, and desalted by PD10 after reconstitution of the apo- form with PQQ. Consequently the concentration of PQQ, in the crystallization conditions and due to its very low *K*d, is equal to the concentration of the enzyme. Holo sGDH were both stored at −80°C in a 50 mM TRIS buffer (pH 7.5) containing 3 mM CaCl_2_.

### Crystallization

Crystals of the holo-sGDH (around 10 mg/ml) from *A. calcoaceticus* were grown with the vapor diffusion hanging drop method at 20°C from 20% (w/v) PEG 6000, 100 mM TRIS pH 8.0, 2 mM zinc chloride (JBScreen PACT++ HTS, condition D12) for the wild-type enzyme and 20% (w/v) PEG 6000, 100 mM TRIS pH 8.0, 200 mM lithium chloride (JBScreen Wizard 4, condition G12) for the Y343F and Y343F/D143E mutants enzyme, respectively. Crystals appeared and grew to their final dimensions after 2 to 4 weeks. For all three structures, space groups, unit-cell dimensions and resolution are presented in Supplementary Data S2. In all cases, the crystallographic asymmetric unit contained two monomers of holo-enzyme.

### X-ray diffraction data collection

Statistics for data collection and refinement of all crystals in this study are given in the table in Supplementary Data S1.

### BM07-FIP2

X-ray diffraction data of the holo-sGDH wild-type were collected on the BM07-FIP2 beamline (wavelength: 0.9795 Å) at the European Synchrotron Radiation Facility (ESRF, Grenoble). A single crystal of 250 × 150 × 150 µm^3^ was mounted in a Hampton nylon loop and cooled down in liquid nitrogen after cryoprotection in paratone oil. 1800 diffraction images were recorded at 100 K with 0.2-degree oscillation and 0.2 s exposure per image with an X-ray beam size of 250 × 250 µm^2^ and a photon flux of 6 × 10^11^ ph/sec. Raddose-3D estimates a dose of 0.9 MGy for the entire dataset with these data collection parameters. Diffraction data were processed using the autoPROC pipeline combined with STARANISO [43].

### SOLEIL-PROXIMA 1

The holo-sGDH mutants were collected on PROXIMA-1 beamline [44] (wavelength: 0.979 Å) at Synchrotron SOLEIL (St. Aubin), project number 20211083. Diffraction data from large, single crystals of the mutant were collected at cryogenic temperatures on an Eiger-16M (Dectris GmbH) detector. The crystals were exposed with 10^12^ photons/sec distributed over 40 × 20 µm^2^ over 360° total rotation, with an oscillation of 0.1° and exposure time of 0.1 s per image. These conditions correspond to an overall dose of approximately 2.7 MGy per crystal. Calculations for dose deposition were performed by *RADDOSE*-3D [36].

Indexing and integration were performed with XDS using the XDSME scripts (https://github.com/legrandp/xdsme) [45]. Molecular replacements were performed using PHASER [46] with the PDB entry 1C9U as the search model. Structures were completed and improved in COOT before refinement with PHENIX.REFINE [47,48]. In order to evaluate the ‘early - late’ difference Fourier, data were reprocessed using autoPROC. The modified form of PQQ was drawn using OpenBabel [49] and chemical restraints prepared with GRADE2 (Global Phasing Ltd). Models were then optimized through iterative rounds of refinement and model building. Model quality was validated using MolProbity [50] before deposition in the PDB under the codes, respectively for wild-type, single mutant and double mutant, 8RG1, 8RFK, 8RE0. Crystal packing was examined using PISA [32]. Figures were prepared with PyMOL (version 2.4.0; The PyMOL Molecular Graphics System, Schrödinger, LLC).

### SEC-SAXS Measurements

The SAXS data was collected at the SWING beamline [51] at the SOLEIL synchrotron, France, equipped with an Eiger 4M detector with a sample-to-detector distance of 2 m. All data were collected in SEC-SAXS mode with a 15°C thermalized in-line BioSec3-300 column (Agilent). Both proteins were concentrated to 10 mg/ml before injecting the protein on to the column. Approximately 45 µl of the sample was injected into the column and the flow rate was maintained at 0.3 ml/min. The running buffer is composed of TRIS 50 mM pH 8 and NaCl 150 mM. The initial data processing steps including masking and azimuthal averaging were performed using the program FOXTROT, and the predicted and measured SAXS curves, given in the Supplementary Figure S10, compared using the ATSAS suite [52].

### UV-visible measurements

Spectroscopic UV-visible measurements of the soluble enzyme were performed in the laboratory on a Cary 100 system from Varian, Inc. (Palo Alto, CA), equipped with a Peltier thermostable multicell holder set to 25°C.

Spectroscopic UV-visible measurements of melted crystals were performed at the *ic*OS Lab at the ESRF (Grenoble) [53] using few nanoliters of solution. Measurements were performed at 100 K.

### Kinetic parameters determination

The glucose oxidation activities of sGDH wild-type, Y343F or D143E/Y343F mutants were determined at 37°C by spectrometry to follow the reduction of 2,6-dichlorophenolindophenol (DCIP) at 600 nm, using phenazine methosulfate (PMS) as the primary electron acceptor. The assay mixture (3 ml) contained 20 mM sodium phosphate, pH 7.0, 60 µM DCIP (ε_600_ = 21.6 mM^−1^.cm^−1^), 600 µM PMS, and 0 to 800 mM glucose or 0 to 400 mM maltose. The reaction was started by adding a sufficient volume of the enzyme to obtain a slope between 0.05 and 2 abs/min at 600 nm. Glucose and maltose solutions were allowed to mutarotate to the anomeric equilibrium for one day before use in kinetic experiments.

The different data were fitted to the following models to take substrate inhibition and negative cooperativity into account according to equation 1 (eqn 1) or inhibition by excess of substrate according to equation 2 (eqn 2) or Michaelis–Menten equation (eqn 3):

(1) $$k_{\text{ss}}=\frac{{\left[S\right]}^{2}\times k_{\text{cat}2}+\left[S\right]\times k_{\text{cat}1}\times K_{M2}}{\left({\left[S\right]}^{3}/K_{I}\right)+{\left[S\right]}^{2}+\left[S\right]\times K_{M2}+K_{M1}\times K_{M2}}$$

(2) $$k_{ss}=\;\frac{k_{cat}\times [S]}{K_{M}+[S]\times (1+([I]/K_{I}))}$$

(3) $$k_{ss}=\;\frac{k_{cat}\times [S]}{K_{M}+[S]}$$

where *k*ss stands for the steady-state rate constant of the enzyme or equivalent to velocity divided by total enzyme concentration. This test was also used to monitor the glucose oxidation activity of the enzyme with 50 mM glucose.

### sGDH sensitivity to H_2_O_2_

The H_2_O_2_ sensitivity of the wild-type enzyme and of the Y343F or D143E/Y343F mutants’ enzymes was monitored between 30 min to 1 h at 25°C after the addition of 25 mM H_2_O_2_. Approximately 8 µN of enzyme were used in 50 mM TRIS pH 7.5 or 50 mM sodium acetate pH 5.0. Data were fitted to a decreasing exponential, according to:

$${\text{Absorbance}}_{340\;\text{nm}}=\;A*e^{-k_{obs}(t-t_{0})}$$

where *A* stands for the magnitude, *k*_obs_ represents the rate constant observed, and *t* represents the time.

### Hydrogen peroxide production

The production of hydrogen peroxide (H_2_O_2_) by sGDH wild-type, Y343F or D143E/Y343F mutants was determined by using *Rb*MPO [54] and a fluorescent probe on a SpectraMax® iD3 Multi-Mode Microplate Reader (Molecular Devices). Measurements were performed in Greiner 384-well clear-bottom (flat-bottom) plates with a reaction volume of 20 µl. In presence of H_2_O_2_ and NaCl at 500 mM, *Rb*MPO at 1 µM converts H_2_O_2_ into HOCl which reacts with APF at 10 µM to release fluorescein (λ_exc_ = 485 nm, λ_emi_ = 525 nm) in sodium phosphate buffer 50 mM pH 7.5 and at 37°C. Several experiments have been done, with or without 250 mM glucose, 25 µM PQQ, 26.5 µM sGDH wild-type, 16.7 µM sGDH Y343F, 24.8 µM sGDH D143E/Y343F or 0.2 µM of *An*GOx. All tests were done in triplicate. Fluorescence was measured after 1 h of reaction and was converted into a quantity of fluorescein from standard fluorescence range (2950000 intensity of fluorescence per pmoles of fluorescein).

### Enzyme aging experiments with or without catalase

For holo-sGDH wild-type, Y343F, and D143E/Y343F mutants, the diminution in absorbance at 340 nm (characteristic wavelength of unmodified PQQ), and the loss of sGDH activity over time permits to assess of the state of aging of the enzyme at 25°C.

Measurements of the absorbance decrease at 340 nm were carried out with 165 µN in site of sGDH in 50 mM TRIS pH 7.5, with or without 287 U/ml of catalase. All assays have been done in triplicate.

Measurements of the loss of sGDH activity over time were carried out as described above for PMS/DCIP test. Approximately 10 µl of sGDH were incubated, with or without 2.87 U catalase (1 U corresponds to the conversion of 1 µmol H_2_O_2_/min) at 25°C, and 25 ng/ml of enzymes were used per test. The steady-state rate constant, *k*ss (s^−1^), characterizes the enzymatic activity.

### Electrochemical experiments

Modified electrodes were prepared as previously reported [55] and were composed of 55 wt% of PAA-PVI-[Os(4,4ʹ-dimethyl-2,2ʹ-bipyridine)2Cl]^+/2+^, 35 wt% of the enzyme and 10 wt% of PEGDGE for a total loading of 108 µg.cm^−2^. Electrochemical characterization of the modified electrodes was performed using a CHI potentiostat CHI760C in a classical 3-electrode set up in a 50 mM TRIS buffer solution (pH 7.5) containing 50 mM glucose under air at +0.3V vs. Ag/AgCl and 25°C in the presence or absence of 10 000 U catalase.

## Supplementary Material

Supplementary Figure S1-S10 and Tables S1-S2

## Data Availability

The structural data are available on protein data bank. The biochemical data presented in this study are available on request from the corresponding authors. The data are not publicly available due to the unavailability of a server dedicated to this task.

## Abbreviations

*An*GOx: glucose oxidase from *Aspergillus niger*
APF: aminophenyl fluorescein
DCIP: 2,6-dichlorophenolindophenol
DTNB: dithionitrobenzoate
GDH: glucose dehydrogenases
GOx: glucose oxidase
PMS: phenazine methosulfate
PQQ: pyrroloquinoline quinone
*Rb*MPO: *Rhodopirellula baltica*
sGDH: soluble glucose dehydrogenase
