# Supplementary material for: Does Acinetobacter calcoaceticus glucose dehydrogenase produce self-damaging H2O2?
Source: Biosci Rep. 2024 May 24;44(5):BSR20240102. doi: 10.1042/BSR20240102 (PMC11130540; doi:10.1042/BSR20240102)
Supplement: Supplementary Figure S1-S10 and Tables S1-S2 [file BSR-2024-0102_supp.zip › BSR-2024-0102_supps.pdf]

Does Acinetobacter calcoaceticus glucose dehydrogenase produce self-damaging H_2O_2?

Supplementary data

Table S1: Data collection and structure refinement statistics. The crystals grew in space group *P*2_1 and structure determination was performed using the 1C9U coordinates as a search model for molecular replacement. The obtention of high resolution data (1.2 - 1.5 Å) allowed us to relax non-crystallographic symmetry constraints during the later cycles of refinements. The resulting structures are all very similar and closely resemble those of Oubrie *et al.* (references 1, 2, 3).

	**WT**	**Y343F**	**D143E/Y343F**
**Data Collection**			
	ESRF FIP2	SOLEIL PX1	SOLEIL PX1
**Space Group**	P2_1	P2_1	P2_1
**Cell dimensions a,b,c(Å) α,β,γ (°)**	60.35,92.51,84.71 90,105,90	60.73, 92.61, 85.34 90, 105.24.90	60.97, 93.04, 85.68 90.0, 105.25. 90
**Wavelength (Å)**	0.9795	0.9786	0.9786
**Isotropic Resolution (Å)**	1.18	1.64	1.32
**Estimated ellipsoidal resolution*** (Å)	1.43,1.31,1.18	1.61,1.5,1.43	1.32, 1.31, 1.25
**R_{pim}**	2.3(59.9)	5.5(46.6)	4.6(55.7)
**R_{merge}**	5.5 (110.3)	7.7(66.0)	4.6(60.6)
**I/σ(I)***	16.5(1.8)	12.9(1.4)	12.7(1.7)
**$CC_{1/2}$***	99.9(65.9)	99.5(46.8)	99.9(84)
**Isotropic Completeness(%)***	69.3(98.7,60.2)	99.8(99.9, 98.0)	95 (99.9, 57)
**Multiplicity**	6.7(6.4)	5.7(3.8)	6.3(3.8)
**Refinement**			
**Refinement Resolution (Å)**	1.19	1.57	1.48
**R_{work}/R_{free} (%)**	14.8/17.27	16./18.34	16.66(20.86)
Model			
**# Protein atoms**	7082	7089	7093
**# Ligand atoms**	66	65	64
**# Waters**	1383	1323	1242
Geometry- RMS deviations			
**Bond lengths (Å)**	0.011	0.010	0.003
**Bond Angles (°)**	1.07	0.97	0.75
**PDB Code**	8RG1	8RFK	8REO

* X-ray diffraction data collection statistics. The average values are followed by, in brackets, the value in the highest resolution shell, except in the case of completeness, where the average value (spherical completeness to highest resolution limit) is followed by the value in the lowest and highest resolution shells (anisotropic completeness). The anisotropic diffraction limits along the crystallographic axes were calculated using the program STARANISO (reference 4)

Table S2: Steady-state kinetics parameters of sGDH wild-type and mutants (Y343F and D143E/Y343F) for glucose and maltose oxidation

		K_{M1} (mM)	K_{M2} (mM)	k_{cat1} (s^{-1})	k_{cat2} (s^{-1})	k_{cat1}/K_{M1} ($mM \cdot s^{-1}$)	k_{cat2}/K_{M2} ($mM \cdot s^{-1}$)	K_I (mM)
Wild-type	Glucose	1.73 ± 1.01	240 ± 70	1.17E+03 ± 0.32E+03	2.58E+04 ± 0.49E+04	673 ± 580	108 ± 52	83 ± 19
	Maltose	46 ± 23	358 ± 147	300 ± 496	6.53E+03 ± 1.21E+03	6.5 ± 14.0	18 ± 11	358 ± 147
Y343F	Glucose	1.2 ± 0.7	499 ± 347	1.45E+03 ± 0.36E+03	6.77 E+04 ± 3.93 E+04	1.20E+04 ± 0.10E+04	136 ± 173	29.5 ± 18.6
	Maltose	10.2 ± 5.4	180 ± 102	3.05E+03 ± 1.22E+03	1.30E+04 ± 2. 29E+04	300 ± 279	73 ± 54	271 ± 87
D143E/Y343F	Glucose	na	290 ± 76	na	8.80E+03 ± 1.71E+03	na	30 ± 14	318 ± 96
	Maltose	na	405 ± 24	na	2.29E+03 ± 0.08E+03	na	5.7 ± 0.5	na

na : non applicable

Figure S1: The cis-peptides present at the dimeric interface. One of the cis-peptide bond (light blue), between L324-Y325, is right next to the P336-Y343 loop, containing the disulfide bridge (yellow). The side chain of Y325 forms a hydrophobic pocket with the aromatic residues F9 and F382. The other cis-peptide, between W265-P266, is present in the 4CD loop (red).

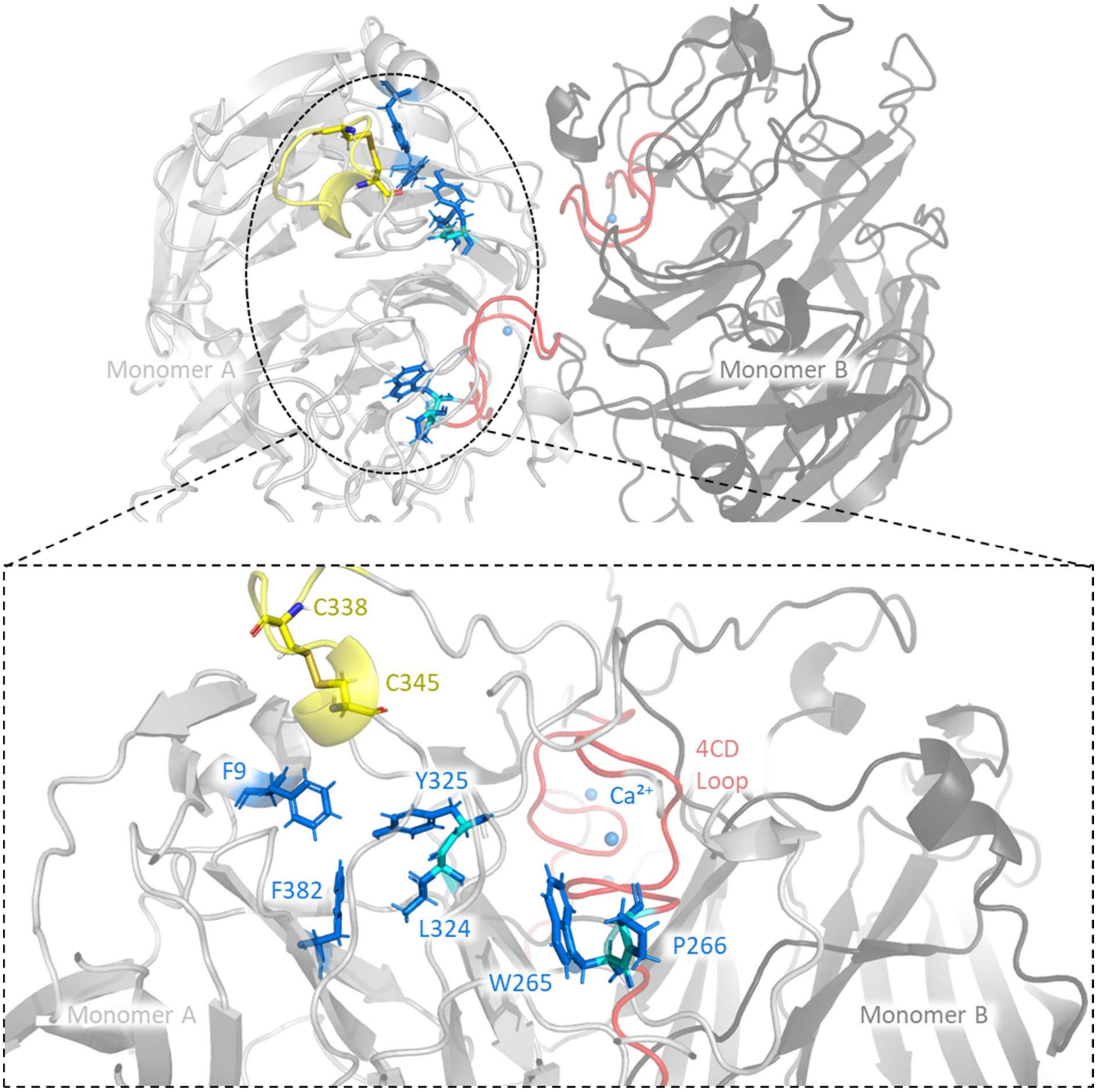

Figure S2: (A) Chemical structure of PQQ and $PQQH_2$. (B) Absorbance spectra of PQQ (red line) and its reduced form, $PQQH_2$, after addition of 1 mM $NaBH_4$ (blue line).

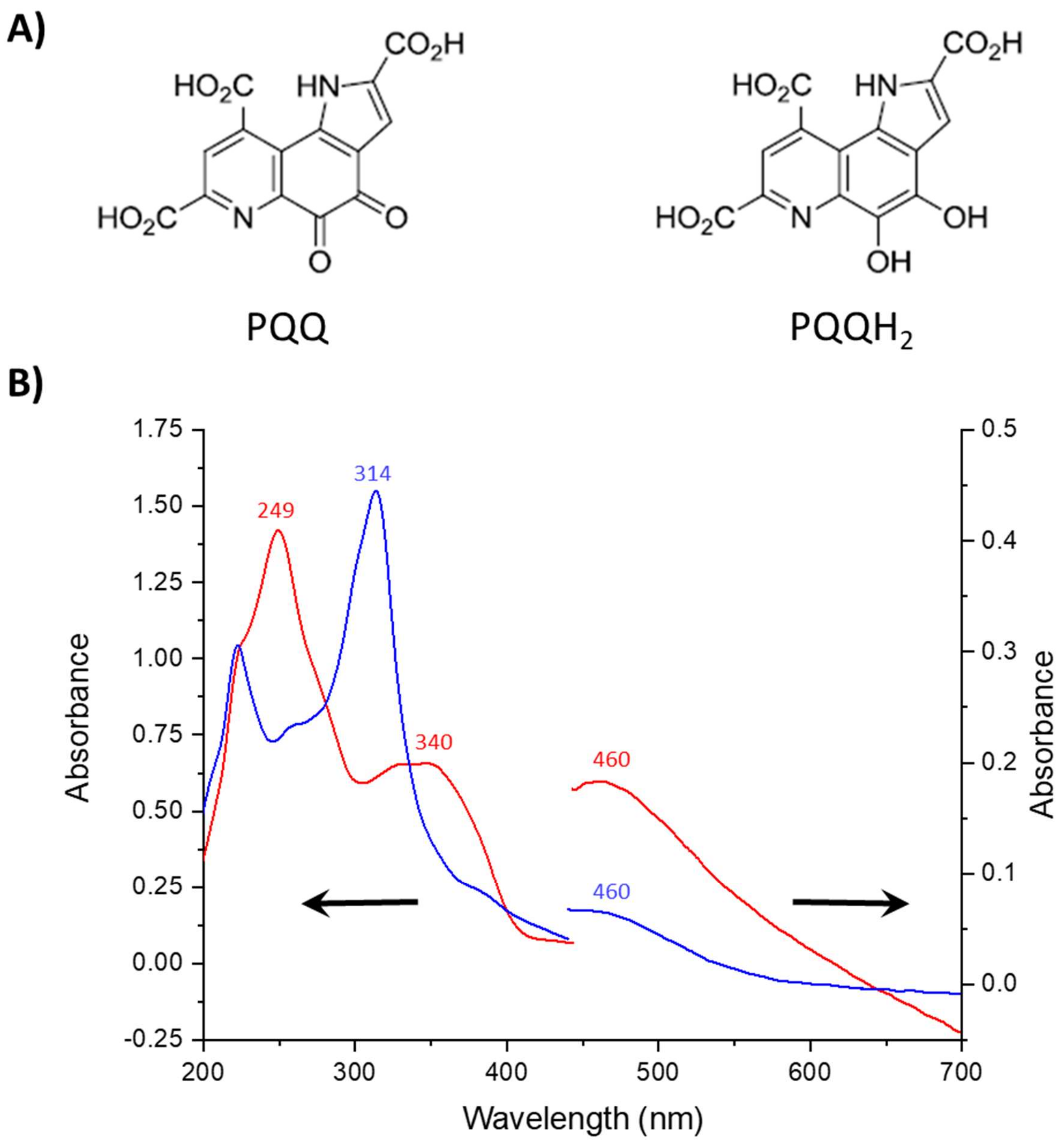

Figure S3: UV-visible spectra of Y343F (A) and D143E/Y343F (B) mutants of sGDH. Spectra were carried out on fresh enzymes (red curves) or on melted crystals that were never exposed to X-rays (black curves) under cryo conditions. The spectrometry measurements were performed at the *ic*OS Lab at the ESRF in Grenoble. Note the disappearance of the peak at 340 nm, characteristic of an intact PQQ, for the crystallized mutants.

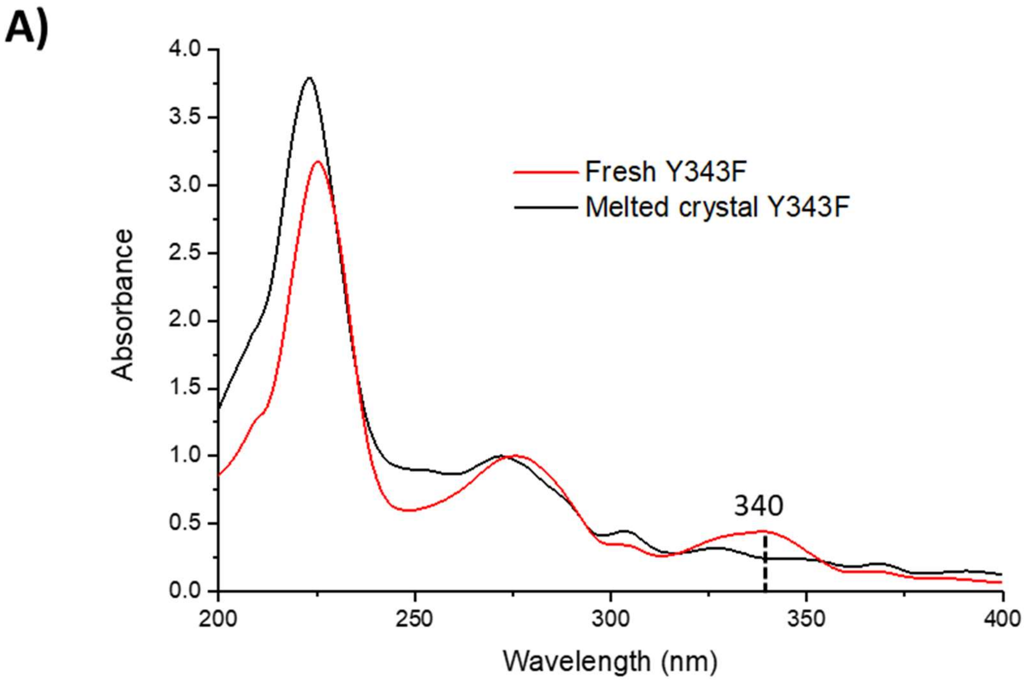

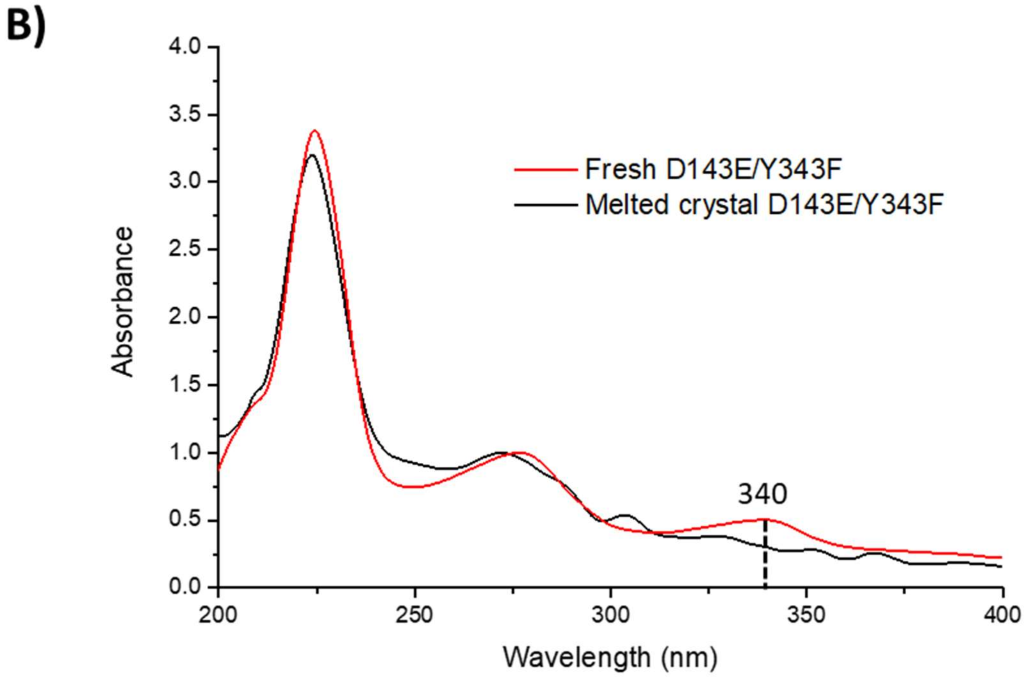

Figure S4: (A) ^{1}H NMR spectra of PQQ at 25°C in d6-DMSO. 1 mg of PQQ (Methoxatin disodium salt, 80198 Sigma) dissolved in 1 mL d6-DMSO containing 1% (v/v) TetraMetylSilane. Spectra was carried out on a JEOL 400 spectrometer (400 MHz) and corresponds to the addition of 100 scans. The chosen analysis window is between 7 and 15 ppm, containing the three expected chemical shifts of PQQ: 6.96 ppm (d), 8.54 (s) and 13.1 (s). The abbreviations used to designate the multiplicity of signals are: s for singlet and d for doublet. (B) Electrospray ionization spectra of PQQ. PQQ (1 mg) was dissolved in 1 mL of methanol. The PQQ ionizes into a mixture of PQQ with the ion $[M-H]^-$ at 329 m/z and the PQQ + 32 m/z which gives the ion $[M-H]^-$ at 361 m/z (red boxes). The hemiacetal methyl adduct corresponding to the additional 32 Da, is due to the reactivity of C5 of PQQ. The mass of the PQQ of formula $C_{14}H_5N_2O_8$ is as expected.

A)

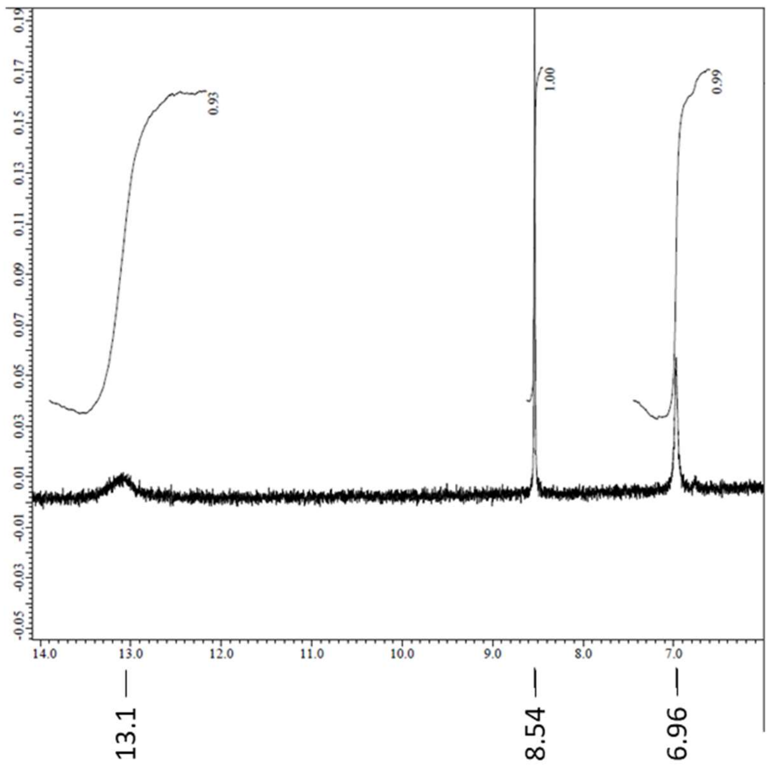

B)

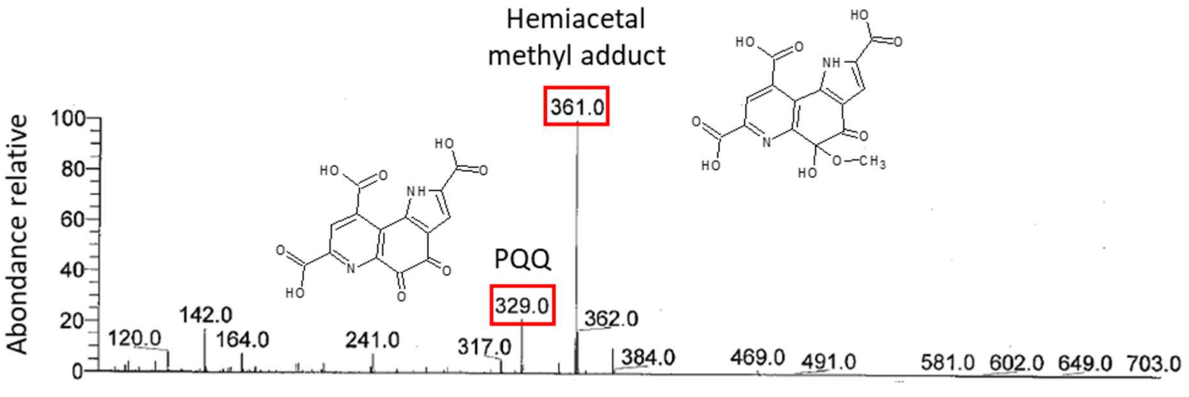

Figure S5: F_{early}-F_{late} difference Fourier synthesis showing $2F_{obs}$-F_c density, from the double mutant protein, displayed at 1.5 σ (grey grid) and difference density at 3 σ (green and red grid for positive and negative peaks) using the program COOT and calculated using the autoPROC and BUSTER programs from Global Phasing. No significant difference in density is visible around the cleaved PQQ apart from minor shifts of individual atoms (for example Ca^{2+}). Similar lack of difference density was observed for the PQQ in both monomers and in all structures from this study.

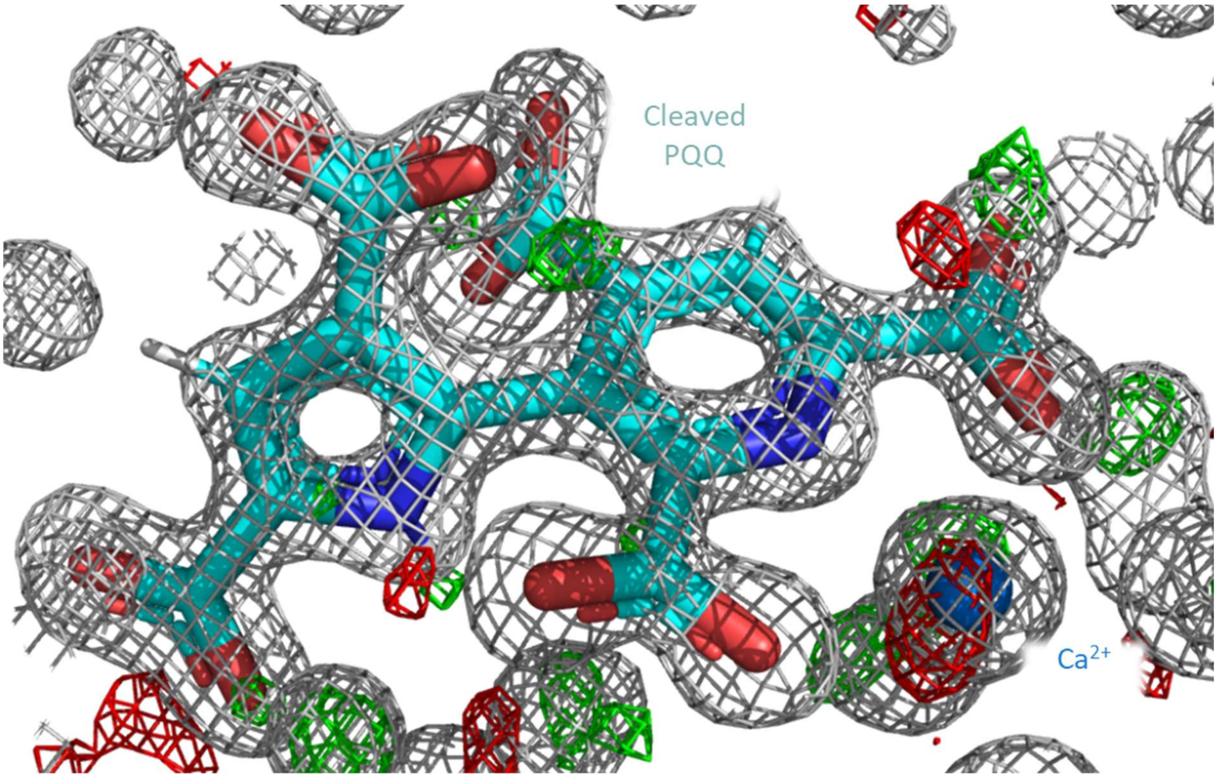

Figure S6: (A) Active site superimposed of the holo-Y343F (blue) and holo-D143E/Y343F (green) mutants of sGDH. The active site of PQQ from the mutant enzymes at pH 8. The electron density at 5 σ from a Polder Omit Map (PHENIX) is superimposed onto a model of cleaved PQQ. The pyrrole ring is seen to be almost perpendicular to the quinone, like wild-type enzyme. (B) PQQ of wild-type enzyme recorded on a rotating anode (0.1 MGy).

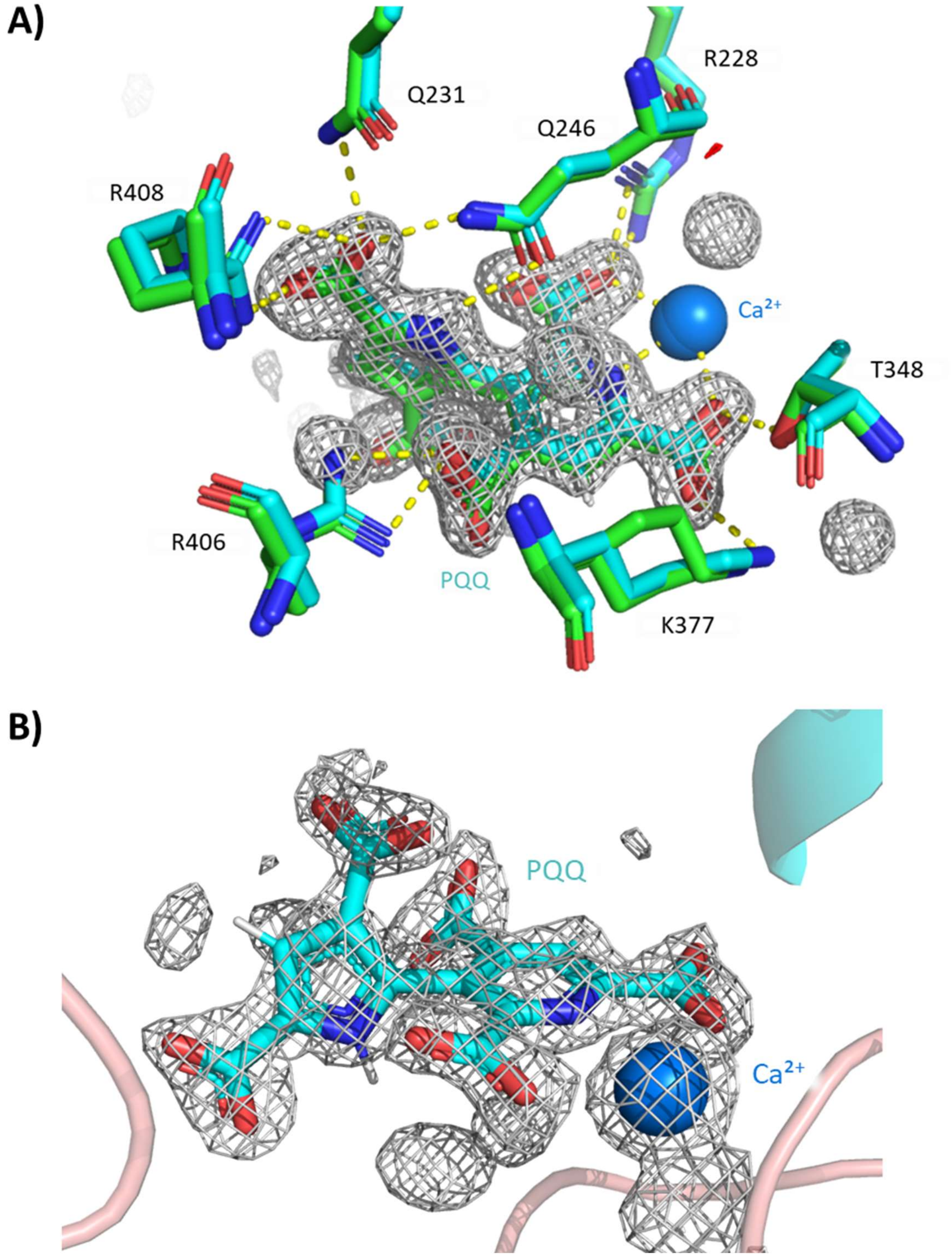

Figure S7: Effect of H_2O_2 on holo-sGDH (wild-type, Y343F mutant and D143E/Y343F mutant) oxidized (A) or reduced (B), at pH 5.0 (1) or at pH 7.5 (2). Absorption spectra were recorded using 8 µN of holo-sGDH (wild-type, Y343F or D143E/Y343F) in 50 mM TRIS/HCl pH 7.5 or 50 mM sodium acetate pH 5.0 (black curves). 25 mM of H_2O_2 (blue curves) were added on oxidized holo-sGDH ($holo_{RED}$-sGDH) or on reduced holo-sGDH ($holo_{RED}$-sGDH) with 5 µM of D-glucose (red curves). The absorption variations were followed over 30 minutes to 1 hour at 25°C.

1) At pH 5.0

(A) Adding H_2O_2 on $Holo_{OX}$-sGDH

(B) Adding H_2O_2 on $Holo_{RED}$-sGDH

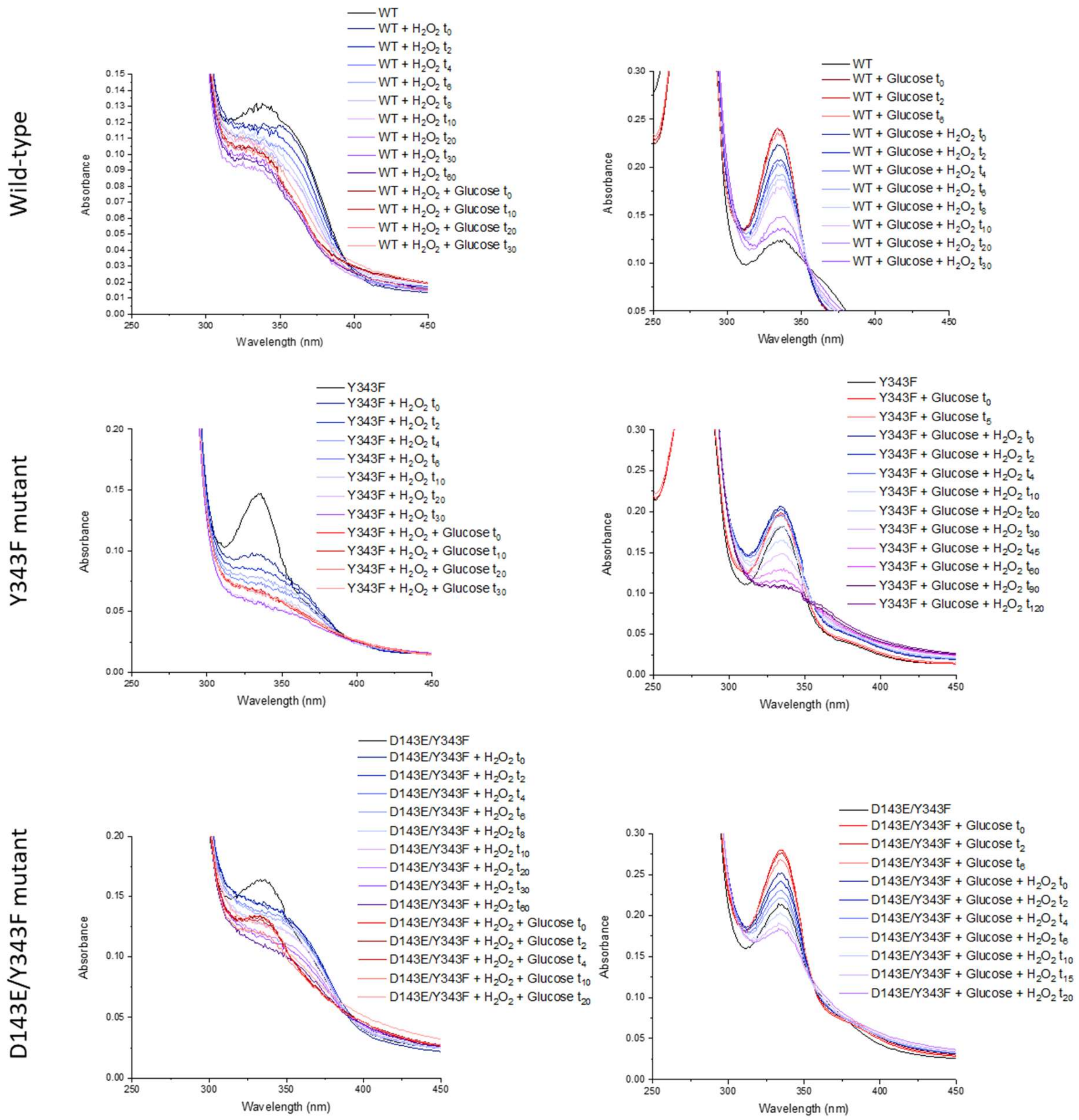

2) At pH 7.5

(A) Adding H_2O_2 on $Holo_{OX}$-sGDH

(B) Adding H_2O_2 on $Holo_{RED}$-sGDH

Wild-type

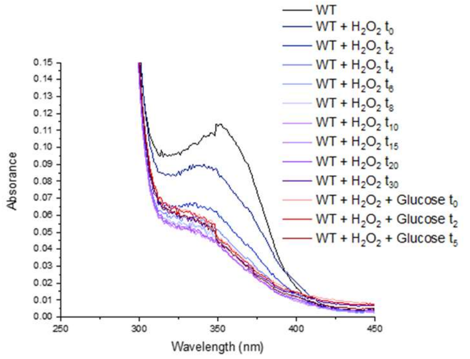

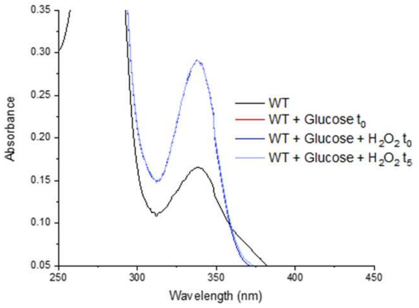

Y343F mutant

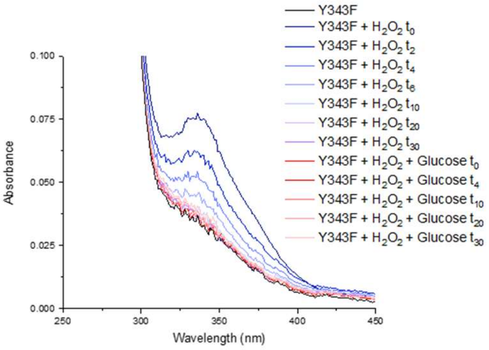

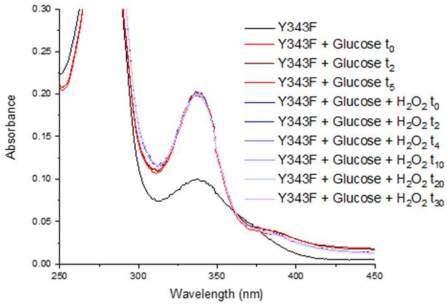

D143E/Y343F mutant

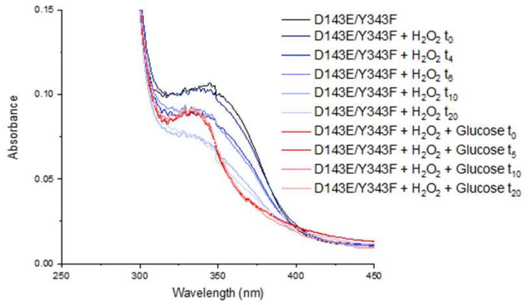

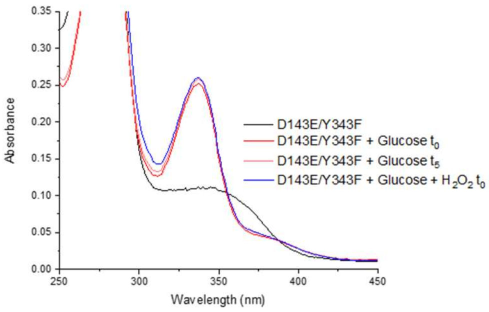

Figure S8: Effect of H_2O_2 on PQQ oxidized (1) or reduced (2), at pH 5.0 or at pH 7.5. Absorption spectra were recorded using 10 µM of PQQ in 50 mM TRIS pH 7.5 or 50 mM sodium acetate pH 5.0 (red curves). 25 mM of H_2O_2 (blue curves) were added on oxidized PQQ (PQQ_{OX}) or on reduced PQQ (PQQ_{RED}) with 1.25 mM of Dithiothréitol (DTT) (black curves). The absorption variations were followed at 25°C until stabilization.

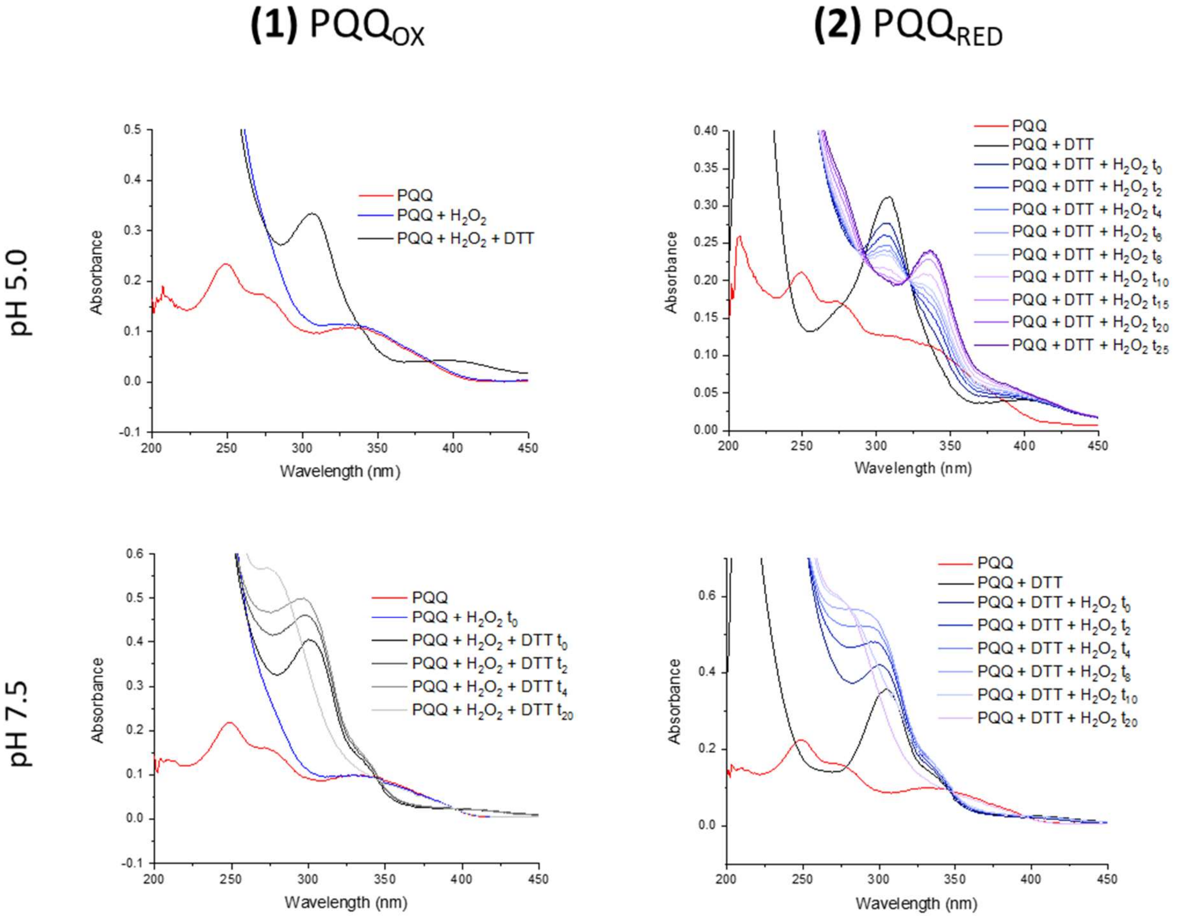

Figure S9: Assay of free cysteines for holo-sGDH Wild-type aged. (A) DTNB (5,5′-Dithio-bis-(2-nitrobenzoic Acid)) was used to quantify thiol group in the sample. Formation of di-anion TNB^{2-} (ε_{412} = 14.15 $mM^{-1} \cdot cm^{-1}$) is proportional to free cysteines and is followed by the increase of absorbance at 412 nm. (B) The enzyme was incubated at 25°C in 50 mM TRIS/HCl pH 7.5 + 3 mM $CaCl_2$ (storage buffer) and the state of the disulphide bridges were followed over time (0, 2, 4, and 8 hours). For experiment, 7.1 µN of sGDH were adding to 0.5 mM TRIS/HCl pH 8.8 containing 2 % SDS and 710 µM DTNB. The maximum expected absorbance delta at 412 nm was 0.2 if the two cysteine per monomer of sGDH was under reduced forms. It seems that the disulfide bridge was not broken at least during the first 8 hours of incubation at 25°C.

A)

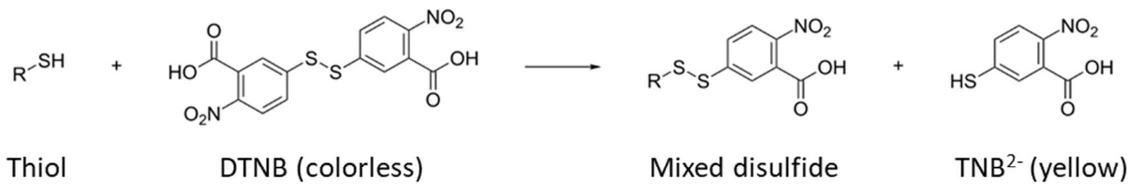

B)

	Absorbance at 420 nm
t_0	0.026
t_2	0.05
t_4	0.027
t_8	0.036

Figure S10: SAXS curves of sGDH proteins (WT, Y343F and D143F-Y143F in black, blue and red respectively). In circles the experimental curves and in line the Crysol fit (based on the refined structures as per Table S2) for each with a chi2 of (5.8, 1.3 and 6.4). The I(q) values has been shifted for a better representation.

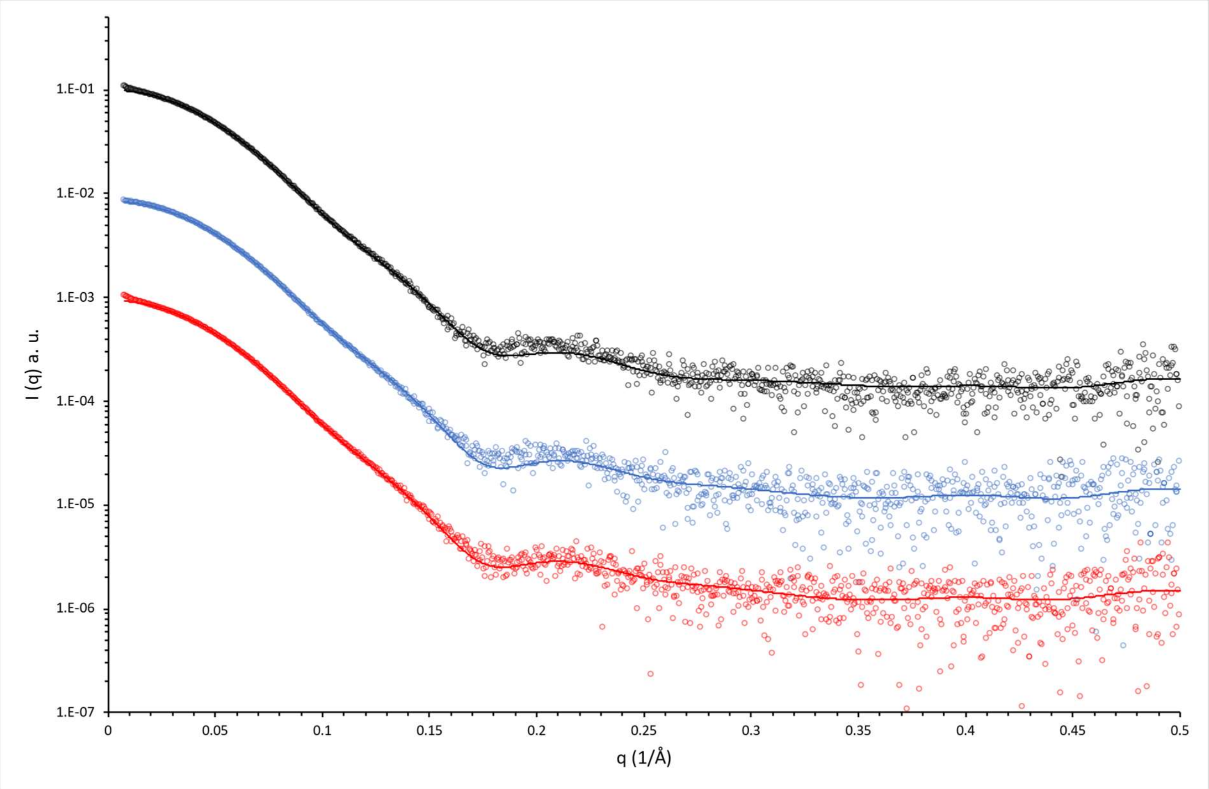

Supplementary Movies.

All the following movies were made with PyMol (reference 5).

Movie 1

The cleaved molecule (pyrrolo-pyridine) of PQQ as seen in the wild type structure (8RG1). The sGDH crystal structure is of a dimer, and the cofactor from the B chain is shown in a ball-stick model. Omit map density (a PHENIX polder map contoured at 5 σ) is shown as a grey mesh, and the surrounding protein chain by a ribbon diagram and the Calcium atom in dark blue. The rotation of the pyrrole group is clearly visible.

The region surrounding the co-factor is not identical in the A and B chains of the dimer. The A chain region tends to be better ordered and the electron density more clear. Several blobs of density, that we have not attempted to interpret, surround the co-factor in the B chain.

Movie 2

A second view of the cleaved PQQ, this time taken from B chain in the double mutant structure 8REO. The color coding is as above. In this movie, a putative molecule of H202 has been modeled (red ball and stick. Since we have no direct evidence, apart from the goodness of fit, that the electron density corresponds to a molecule of H_20_2, this density is left out of the refined model deposited in the PDB.

Movie 3

This movie shows a PyMol representation, prepared by the authors, of the dimeric structure of sGDH as deposited in the PDB by Oubrie (1C9U, A. Oubrie et al, EMBO Journal (1999) 18, 5187-5194). PQQ (uncleaved) is shown as a cyan ball and stick model, sGDH as a ribbon drawing and the Calcium sites as dark blue spheres. Coordinates and structure factors were downloaded from PDB-Redo (Joosten et al, reference 6).

References

1. Oubrie A. Structure and mechanism of soluble glucose dehydrogenase and other PQQ-dependent enzymes. Biochim Biophys Acta BBA - Proteins Proteomics 2003;1647(1):143–151; doi: 10.1016/S1570-9639(03)00087-6.

2. Oubrie A, Rozeboom HJ, Kalk KH, Duine JA, Dijkstra BW. The 1.7 Å crystal structure of the apo form of the soluble quinoprotein glucose dehydrogenase from *Acinetobacter calcoaceticus* reveals a novel internal conserved sequence repeat. J Mol Biol 1999;289(2):319–333; doi: 10.1006/jmbi.1999.2766.

3. Oubrie A, Rozeboom HJ, Dijkstra BW. Active-site structure of the soluble quinoprotein glucose dehydrogenase complexed with methylhydrazine: A covalent cofactor-inhibitor complex. Proc Natl Acad Sci U S A 1999;96(21):11787–11791; doi: 10.1073/pnas.96.21.11787.

4. Tickle IJ, Sharff A, Flensburg C, Smart O, Keller P, Vonrhein C, *et al*. STARANISO Anisotropy & Bayesian Estimation Server. n.d. Available from: https://staraniso.globalphasing.org/cgi-bin/staraniso.cgi [Last accessed: 6/30/2023].

5. Schrödinger L, DeLano W. PyMOL [Internet]. 2020. Available from: http://www.pymol.org/pymol

6. Joosten RP, Long F, Murshudov GN, Perrakis A. The PDB_REDO server for macromolecular structure model optimization. IUCrJ 2014;1(Pt 4):213–220; doi: 10.1107/S2052252514009324.
